# Macroscopic features of scurvy in human skeletal remains: A literature synthesis and diagnostic guide

**DOI:** 10.1002/ajpa.23699

**Published:** 2018-10-09

**Authors:** Anne Marie E. Snoddy, Hallie R. Buckley, Gail E. Elliott, Vivien G. Standen, Bernardo T. Arriaza, Siân E. Halcrow

**Affiliations:** ^1^ Department of Anatomy University of Otago Dunedin Otago New Zealand; ^2^ Department of Anatomy Ross University School of Medicine Portsmouth Dominica; ^3^ Departmento de Antropología Universidad de Tarapacá Arica Chile; ^4^ Instituto de Alta Investigación, Universidad de Tarapacá Arica Chile

**Keywords:** Atacama Desert, bioarchaeology, Differential diagnosis, human anatomy, metabolic bone disease, paleopathology

## Abstract

The past two decades have seen a proliferation in bioarchaeological literature on the identification of scurvy, a disease caused by chronic vitamin C deficiency, in ancient human remains. This condition is one of the few nutritional deficiencies that can result in diagnostic osseous lesions. Scurvy is associated with low dietary diversity and its identification in human skeletal remains can provide important contextual information on subsistence strategy, resource allocation, and human‐environmental interactions in past populations. A large and robust methodological body of work on the paleopathology of scurvy exists. However, the diagnostic criteria for this disease employed by bioarchaeologists have not always been uniform. Here we draw from previous research on the skeletal manifestations of scurvy in adult and juvenile human skeletal remains and propose a weighted diagnostic system for its identification that takes into account the pathophysiology of the disease, soft tissue anatomy, and clinical research. Using a sample of individuals from the prehistoric Atacama Desert in Northern Chile, we also provide a practical example of how diagnostic value might be assigned to skeletal lesions of the disease that have not been previously described in the literature.

## 1 INTRODUCTION

Scurvy, a condition caused by severe and prolonged vitamin C deficiency, has received increasing attention in the bioarchaeological literature in recent years. This proliferation of research began with the identification by Donald Ortner and colleagues of a suite of dry bone lesions which the authors argue can be intuitively linked to the unique pathophysiology of this disease (Ortner, Butler, Cafarella, & Milligan, 2001; Ortner & Ericksen, 1997; Ortner, Kimmerle, & Diez, 1999). Subsequent researchers have expanded on this work, both by extending Ortner's logical process of lesion identification to osseous changes at other sites in the skeleton and by integration of clinical radiographic and histological features of scurvy (Brickley & Ives, 2008; Geber & Murphy, 2012; Maat, 2004; Mays, 2008; Moore & Koon, 2017; Stark, 2014).

The identification of scurvy in past populations is meaningful for reasons that extend beyond the diagnosis of a pathological condition within the individual. Vitamin C deficiency is a condition associated with low dietary diversity and can be caused by general famine conditions or nutritional dependence on vitamin C poor staple foods, such as cereal crops (Cheung et al., 2003; World Health Organization, 1999). As such, identification of scorbutic individuals in the archeological record can contribute significantly to studies of the paleoenvironment, subsistence transitions such as agricultural intensification, and even cultural practices involving resource allocation and social inequality (Buckley et al., 2014; Crandall, 2014; Snoddy, Halcrow, Buckley, Standen, & Arriaza, 2017). Although many studies have contributed to methodological development (Brickley & Ives, 2006, 2008; Buckley et al., 2014; Maat, 2004; Ortner et al., 1999, 2001), and theoretical frameworks for the study of scurvy in the past have begun to emerge (Armelagos, Sirak, Werkema, & Turner, 2014; Stark, 2014), a comprehensive literature synthesis and diagnostic guide for the identification of scurvy in humans is still needed. This article provides a review of the paleopathological literature on the diagnosis of scurvy in adult and juvenile (0–15 years) human skeletal remains, a critical evaluation of the clinical basis of diagnostic macroscopic skeletal lesions, and a case‐study demonstrating a new approach to the establishment of diagnostic features is presented. Finally, a new weighted diagnostic system is presented for use in the differential diagnosis process.

## 2 SCURVY, DIETARY DIVERSITY, AND PALEODIETARY RECONSTRUCTION

Nutritional deficiencies are notoriously difficult to assess from skeletal pathology. This is because most of these conditions (e.g., niacin deficiency, protein‐energy malnutrition) either do not leave any osseous markers or do not result in a pathognomonic suite of bony lesions (Ortner, 2003; Oxenham & Cavill, 2010; Paine & Brenton, 2007; Walker, Bathurst, Richman, Gjerdrum, & Andrushko, 2009). Traditionally, bioarchaeologists have relied upon paleofaunal and paleobotanical remains, as well as non‐specific markers of physiological disruption in bone and teeth (e.g., Harris lines, linear enamel hypoplasia), to provide contextual evidence of dietary stress in past populations (Cohen & Armelagos, 1984; Cohen & Crane‐Kramer, 2007; Huss‐Ashmore, Goodman, & Armelagos, 1982). Advances in isotopic analysis of human remains have allowed the generalized reconstruction of trophic relationships and resource use in ancient human groups (Hedges & Reynard, 2007; Schoeninger, 2014). However, these analyses cannot provide direct information on the intake of individual micronutrients, such as vitamins and minerals, many of which are essential for maintaining basic metabolic function. As such, the identification of specific diseases of poor micronutritional intake in skeletal remains has the potential to contribute significantly to the creation of a holistic portrait of health, resource use, and human‐environmental interactions in the ancient past (Buckley et al., 2014; Snoddy et al., 2017; Stark, 2014; Wilbur, Farnbach, Knudson, & Buikstra, 2008).

Micronutrient malnutrition disorders, such as vitamin C deficiency, rarely occur in isolation and because of this the identification of scurvy in ancient human remains can be an important proxy for population‐wide nutritional status (Snoddy et al., 2017; Stark, 2014). As discussed above, scurvy is associated with low dietary diversity, and can be found in individuals experiencing general undernutrition (e.g., starvation) or those relying on a few, micronutrient poor staple foods for caloric sufficiency (Snoddy et al., 2017; World Health Organization, 1999). Clinically, individuals suffering from scurvy are also usually deficient in a number of other micronutrients, such as folate or iron, which will not necessarily leave diagnostic osseous markers (Pimentel, 2003; Popvich, McAlhany, Adewumi, & Barnes, 2009). The development and consistent application of a robust diagnostic protocol for the identification of scurvy from human skeletal remains is therefore of importance to those who study the wider context of nutritional stress in ancient human groups.

## 3 VITAMIN C IN HUMAN HEALTH

The role of vitamin C in human physiology has been reviewed in detail elsewhere (Levine & Padayatty, 2014), but a brief overview is presented here. Ascorbic acid functions as a reducing agent within the body, catalyzing enzymatic reactions and acting as a potent antioxidant by pairing with free radicals (Levine & Padayatty, 2014, p. 400). Most mammals have the ability to endogenously synthesise ascorbic acid from glucose. However, primates, including humans, lack the hepatic enzyme gluconoactone oxidase, which is necessary for the synthesis of the ascorbic acid precursor (Drouin, Godin, & Page, 2011). These animals must therefore meet their physiological need through dietary intake of substances that are rich in pre‐synthesized ascorbic acid (Du, Cullen, & Buettner, 2012, p. 444). All photosynthetic plants have the ability to synthesize ascorbic acid (Smirnoff & Wheeler, 2000). Fruits and dark green vegetables, particularly citruses, asparagus, broccoli, and kale, are rich sources of vitamin C (Levine, Rumsey, Daruwala, Park, & Wang, 1999, p. 1417). The livers of animals that are capable of endogenous synthesis of ascorbic acid (e.g., most terrestrial vertebrates and marine mammals) are also rich in this nutrient and can be an important complementary source of vitamin C for humans in environments where vegetation is scarce (Fediuk, Hidiroglou, Madère, & Kuhnlein, 2002; Geraci & Smith, 1979).

The recommended daily allowance (RDA) of vitamin C for adult (>19 years) males is 90 mg and 75 mg for adult females, with an increased metabolic need during pregnancy (80–85 mg), lactation (115–120 mg), wound healing, and infectious illness (Hunt, Chakravorty, Annan, Habibzadeh, & Schorah, 1994; NIH, 2018; Ringsdorf & Cheraskin, 1982). Children vary considerably in their daily requirement of vitamin C. The RDA for infants 0–12 months is unestablished, although 40‐50 mg is clinically recommended as a precautionary measure (NIH, 2018). The RDA for children aged 1–3 years is 15 mg, with incremental increases until reproductive maturity is reached (NIH, 2018). The absolute minimum daily intake necessary to prevent scurvy is thought to be 10 mg in adults (Hirschmann & Raugi, 1999: 899; Levine et al., 1999; Pimentel, 2003, p. 331; Popvich et al., 2009). If this minimum requirement is unmet long enough for somatic stores to fall below 350 mg (~1–3 months), scurvy can occur and if left untreated it is invariably fatal (Fain, 2005; NIH, 2018).

## 4 CLINICAL MANIFESTATIONS OF DEFICIENCY

Scurvy is associated with a number of nonspecific symptoms, such as fatigue, irritability, and depression (Olmedo, Yiannias, Windgassen, & Gornet, 2006; Pimentel, 2003; Popvich et al., 2009). However, the majority of clinical signs are directly related to defective type II collagen synthesis. Ascorbic acid acts as an electron donor to two enzymes involved in the hydroxylation of the amino acids lysine and proline. This hydroxylation process is necessary for the stabilization of the final triple helix structure of mature collagen, and if the structural integrity of collagen is compromised, widespread damage of connective tissues can occur (Hirschmann & Raugi, 1999 p. 899). The extracellular matrix of bone is largely composed of collagen (Boskey & Robey, 2013) and poor or non‐existent osteoid formation is a hallmark of ascorbic acid deficiency, with the rapidly developing juvenile skeleton typically more severely affected than that of the adult (Fain, 2005; Joffe, 1961). Both adults and juveniles may exhibit generalized osteopenia and cortical thinning due to the cessation of osteoid formation, but this feature is not strongly diagnostic (Fain, 2005; Joffe, 1961; Weinstein, Babyn, & Zlotkin, 2001; Young, Schiliro, & Russo, 1979). Likewise, subperiosteal hemorrhage can result in a radiographically visible lifting of the periosteum from the underlying cortex. However, subperiosteal new bone is generally only visible following the reintroduction of vitamin C and resumption of osteoid formation and must usually be extensive in order to be radiographically visualized (Brailsford, 1953; Joffe, 1961; Sprague, 1976; Young et al., 1979).

Musculoskeletal lesions occur in the majority of clinical cases of subadult scurvy and radiography of suspected scorbutic individuals is standard practice in differential diagnosis (Pimentel, 2003; Riepe, Eichmann, Oppermann, Schmitt, & Tunnessen, 2001; Verma et al., 2007; Young et al., 1979). The majority of radiographically visualized diagnostic lesions are metaphyseal and associated with defective endochrondral growth. Demineralization of the cortex of metaphyses results in a characteristic radiographic “ground glass” appearance (Jaffe, 1972; Riepe et al., 2001). Alternating radiolucent (Trümmerfeld zone) and radiopaque (White line of Frankel) bands, which represent defective bone matrix formation and poor resorption in the provisional zone of calcification respectively, may be evident in regions of active endochrondral growth (e.g., metaphyses of lone bones, sternal end of ribs) (Jaffe, 1972; Young et al., 1979). Wimberger's ring, a band of increased mineral density around epiphyses, is also caused by poor resorption of calcified cartilage (Jaffe, 1972; Popvich et al., 2009). Clefting of the lateral metaphyses (“corner sign”) may occur as the result of microfractures within the provisional zone of calcification (Riepe et al., 2001). When these microfractures heal, bony spurs may form on the lateral aspect of the metaphyses of lower long bones (Burk & Molodow, 2007). Most of these lesions remodel quite rapidly following reintroduction of vitamin C and are only present in active cases of severe deficiency, although lesions associated with increased mineral density may persist for years (Jaffe, 1972; Sprague, 1976).

One of the tissues most dramatically affected by ascorbic acid deficiency in both adults and children is the basement membrane of blood vessels, which functions in the connection and support of the vascular epithelial wall to the underlying endothelium (Popvich et al., 2009, p. 411; Saladin, 2014, p. 750–752). In scorbutic individuals, compromised integrity of the vascular wall causes widespread, chronic hemorrhage in response to minor trauma and normal muscular action, resulting in perivascular edema, petechia (pin‐point cutaneous bruising), gingival bleeding, and subperiosteal hematomas (Hirschmann & Raugi, 1999; Olmedo et al., 2006; Popvich et al., 2009). Subperiosteal hemorrhage and bleeding into joint spaces (hemarthrosis) can cause intense pain and inflammation, and may lead to compromised mobility (Hirschmann & Raugi, 1999, p. 902). Gingival hemorrhage due to scurvy can cause secondary alveolar bone resorption, resulting in periodontal disease and antemortem tooth loss (Hirschmann & Raugi, 1999, p. 902). It is primarily the secondary effects of this chronic bleeding, particularly in the subperiosteal space of long bones, cranial vault, orbits, small facial bones and the alveolar bone of the mandible and maxilla, and at entheseal sites which is thought to lead to many of the lesions commonly employed by paleopathologists in the differential diagnosis of scurvy from skeletal remains (Brickley & Ives, 2008; Ortner, 2003; section 10).

## 5 THE “ORTNER CRITERIA”: PALEOPATHOLOGICAL INFERENCE USING KNOWN ANATOMICAL RELATIONSHIPS

**Figure 1:**
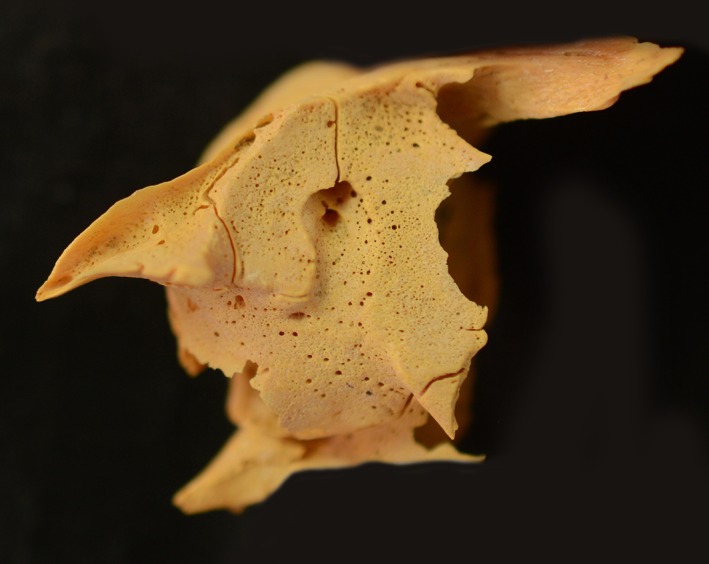
Superior (orbital) surface of the left maxilla of an infant (~9 months, unknown sex) with probable scurvy exhibiting fine, abnormal cortical porosity

**Figure 2:**
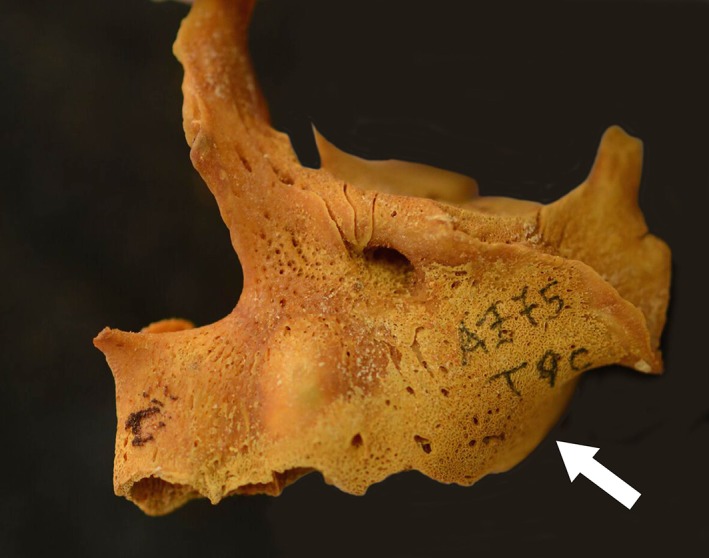
Anterior surface of left maxilla of an infant (1 year ±4 months, unknown sex) with probable scurvy exhibiting active subperiosteal new bone and abnormal porosity (white arrow)

As discussed above, the majority of features employed by bioarchaeologists in the diagnosis of scurvy from skeletal remains have their foundation in the work of Ortner & colleagues. Using both anatomical (Ortner & Ericksen, 1997) and archaeological samples of non‐adult remains (Ortner et al., 1999, 2001) these authors have identified a suite of macroscopic bony lesions which they argue can be intuitively linked to the hemorrhagic effects of scurvy, specifically low‐grade bleeding due to the effects of minor muscular actions—such as masticatory movements—on weakened vasculature. The scorbutic lesions proposed by Ortner et al., as well with their anatomical associations and hypothesized mechanisms of formation, are outlined in Table 1 and described in detail in section ten. The majority of these are cranial, consisting of clustered, fine cortical porosity and are argued to be the result of bleeding of vasculature associated with the muscles of mastication, such as the temporalis and pterygoids (Ortner et al., 1999, 2001; Ortner & Ericksen, 1997). Capillary proliferation is part of the inflammatory response to the presence of extravascular blood; these new vessels act as a delivery system for phagocytic cells of the innate immune system to aid in the removal of hemorrhagic material (Browder, Folkman, & Pirie‐Shepherd, 2000). Ortner & others argue that this hypervascular (angiogenic) response to extravasated blood will result in fine (<1 mm across) abnormal porosity at sites of subperiosteal hemorrhage as new capillaries create pathways through the bony cortex (Klaus, 2017; Ortner et al., 2001; Figure 1). Once sufficient levels of vitamin C are restored for osteoid formation, islands of porous new bone may be deposited at these sites of subperiosteal bleeding (Ortner et al., 1999, 2001; Riepe et al., 2001; Figure 2). Some bioarchaeologists have used this logic to interpret subperiosteal scorbutic lesions as “healing” individuals, although, as Klaus (2017) and others have pointed out, it is likely that relatively little vitamin C is needed for the resumption of osteoid formation and the formation of new bone should not necessarily be interpreted as a full return to health (Klaus, 2017; Stark, 2014).

**Table 1:** Macroscopic osseous lesions that have been attributed to scurvy with potential anatomical associations and diagnostic strength within our method

Lesion location	Lesion type	Anatomical structure: Vasculature	Anatomical structure: Muscle(s)	Action(s)	Paleopathological references	Clinical support	Proposed diagnostic strength
Ectocranial parietal and/or squamous temporal	Abnormal cortical porosity, SPNB	Superficial and deep temporal arteries	Temporalis	Mastication, speech	Ortner & Ericksen, 1997; Ortner et al., 1999; Ortner et al., 2001	Indirect: Barlow, 1883	**Diagnostic**
Endocranial calvarium	Islands of abnormal cortical porosity and/or SPNB	Meningeal arteries (anterior and posterior branches)	NA	NA	Brown & Ortner, 2011; Snoddy et al., 2017	Direct: Barlow, 1883; Indirect: Ingalls, 1936	**Suggestive**
Sphenoid: Greater wing	Abnormal cortical porosity, bilateral SPNB	Anterior deep temporal artery	Temporalis (indirect)	Mastication, speech (various)	Ortner & Eriksen, 1997; Ortner et al., 1999; Ortner et al., 2001	None; anatomically intuitive	**Diagnostic**
Sphenoid: Foramen rotundum	SPNB	Artery of foramen rotundum (branch of maxillary artery)†	Temporalis (indirect)	Vasculature associated with the maxillary branch of the trigeminal nerve (V2)	Geber & Murphy, 2012	None; anatomically intuitive	**Diagnostic**
Sphenoid: Lesser wing	Abnormal cortical porosity	Opthamic vein, middle meningeal artery†	NA	NA	Brickley & Ives, 2006	None; anatomically intuitive	**Suggestive**
Sphenoid: Pterygoid fossae/plates	Abnormal cortical porosity, SPNB	Arteries to the medial and lateral pterygoid muscles (branches of the maxillary artery)	Lateral pterygoid; medial pterygoid (origin of deep head)	Mastication, speech (various)	Crist & Sorg, 2014; Klaus, 2017; This paper	None; anatomically intuitive	**Diagnostic**
Frontal: Orbital roof	Abnormal cortical porosity, SPNB	Ethmoidal, supra‐orbital, supra‐trochlear and lacrimal branches of the ophthalmic artery.	Extrinsic occular muscles (indirect) †	Internal movement of the eye	Ortner & Ericksen, 1997; Ortner et al., 2001; Brickley et al.., 2006; Klaus, 2014	Indirect: Barlow, 1883; Verma et al., 2007	**Diagnostic**
Zygomatic: Lateral aspect	Abnormal cortical porosity; SPNB	Zygomaticoorbital and zygomaticofacial arteries†	Orbicularis occuli (indirect) †	Facial expression (various)	Ortner et al., 1999; Ortner et al., 2001; Snoddy et al., 2017	None; anatomically intuitive	**Suggestive**
Zygomatic: Internal (posterior) aspect	Abnormal cortical porosity	Masseteric branches (maxillary artery), orbital and facial branches of the superficial temporal artery†	Masseter (origin) †; temporalis (indirect)	Facial expression (various)	Ortner et al., 2001	None; anatomically intuitive	**Suggestive**
Maxillae: Anterior surface/infraorbital foramina	Abnormal cortical porosity, SPNB	Infraorbital artery	Buccinator (origin) †; orbicularis ori (indirect) †	Mastication, suckling, speech (various)	Ortner et al., 2001	None; anatomically intuitive	**Diagnostic**
Maxillae: Posterior surface	Abnormal cortical porosity, SPNB	Posterior superior alveolar artery	Medial pterygoid (origin of superficial head)	Mastication, speech (various)	Ortner & Eriksen, 1997; Ortner et al., 1999; Ortner et al., 2001	None; anatomically intuitive	**Diagnostic**
Maxillae: Palatal surface	Abnormal porosity	Maxillary artery (greater palatine branch)	Various masticatory muscles (indirect)	Mastication, speech (various)	Ortner & Eriksen, 1997; Ortner et al., 1999; Ortner et al., 2001	None; anatomically intuitive	**Diagnostic**
Mandible: Medial surface/coronoid process	Abnormal cortical porosity, SPNB	Maxillary artery (masseteric branches)	Temporalis (insertion)	Mastication, speech (various)	Ortner & Eriksen, 1997; Ortner et al., 1999; Ortner et al., 2001	None; anatomically intuitive	**Diagnostic**
Mandible: Mylohyoid line	Bilateral SPNB	Inferior alveolar artery (mylohyoid branch)	Mylohyoid (origin)	Mastication, speech (various)	This paper	None; anatomically intuitive	**Suggestive**
Occipital: Inferior surface of pars basilaris	Abnormal cortical porosity, SPNB	Inferior thyroid artery (ascending cervical branch) †	Longus capitis (insertion)	Flexion of neck	Moore & Koon, 2017; cf. González, Rodríguez, Cambra‐Moo, Pérez, & Martín, 2018	Indirect: e.g., Olmedo et al., 2006	**Suggestive**
Scapula: Supraspinous fossa	Abnormal cortical porosity	Suprascapular artery	Supraspinatus (origin)	Abduction of arm	Ortner et al., 2001	Indirect: Barlow, 1883	**Diagnostic**
Scapula: Infraspinous fossa	Abnormal cortical porosity, SPNB	Suprascapular artery; circumflex branch of subscapular artery	Infraspinatus (origin); teres minor (origin)	Lateral rotation of arm	Ortner et al., 2001; This paper	Indirect: Barlow, 1883	**Diagnostic**
Ilium: Visceral surface	Abnormal porosity, SPNB, VIs	Internal iliac artery (iliolumbar branch)	Iliacus (origin)	Flexion and medial rotation of hip	Brown & Ortner, 2011	Indirect: Barlow, 1883	**Suggestive**
Femur: Linea aspera and surrounding area	SPNB	Femoral artery (profundus branch)	Adductor longus (insertion)	Adduction of femur; flexion of hip	Buckley et al., 2014	None; anatomically intuitive	**Suggestive**
Appendicular skeleton: Diaphyses/ metaphyses	SPNB (diffuse)	Multiple	Multiple	Multiple	Van der Merwe, Steyn, & Maat, 2010; Brown & Ortner, 2011; Geber & Murphy, 2012; Klaus, 2014; Buckley et al., 2014; Snoddy et al., 2017	Direct: Riepe et al., 2001; Noordin, Baloch, Rashid, & Ahmad, 2012	**Diagnostic**
Ribs: Costochrondral junction	Swelling/flaring	NA	NA	NA	Buckley et al., 2014; Schattmann et al., 2016	Direct: Jaffe, 1972	**Suggestive**
Ribs: Antero‐lateral shaft	SPNB	Multiple	Serratus anterior, pectoralis minor	Protraction of the scapulae, adduction of the humerus	Buckley et al., 2014; Snoddy et al., 2017	Indirect: Barlow, 1883	**Suggestive**
Vertebrae: Bodies	Biconcavity, osteopenia	NA	NA	NA	None	Direct: Joffe, 1961	**Suggestive**
Appendicular skeleton	Ossified hematomas: Bilateral	Multiple	Multiple	Multiple	Maat, 2004; Van der Merwe et al., 2010	Limited: Brailsford, 1953	**Suggestive**
Appendicular skeleton	Metaphyseal cupping/flaring	NA	NA	NA	Schattmann et al., 2016	Limited: Sprague, 1976	**Suggestive**

SPNB = subperiosteal new bone. †structure not implicated by referenced author(s) but role is anatomically intuitive. VIs = vascular impressions. NA = not applicable. More than one diagnostic feature is required in a single individual for classification of probable scurvy and at least one diagnostic and multiple suggestive features are required for classification of possible scurvy. Visual examples of each listed lesion are provided in Supporting Information Table S1.

Although the lesions observed by Ortner et al. are not part of the clinical suite of bony changes employed in the differential diagnosis of scurvy, their association with scurvy is logical from an anatomical and pathophysiological perspective. As mentioned above, subperiosteal hemorrhage and hematomas are a documented feature of active scurvy, and subperiosteal new bone following the reintroduction of vitamin C is a classic radiographic finding (Jaffe, 1972; Noordin et al., 2012; Riepe et al., 2001; Weinstein et al., 2001). These hemorrhages are typically found on long bones, particularly in the metaphyseal region of lower limbs, although intraocular and subdural hematomas have also been reported (Ingalls, 1936; Verma et al., 2007). Islands of subperiosteal new bone on the endocranial surface, the metaphyses of the lower limbs, and the orbital roof do therefore have clinical support as features associated with scurvy, although trauma should also be considered in differential diagnosis. Accounts from historic pathologists also provide some support for several of the bony lesions attributed to the hemorrhagic effects of scurvy by Ortner and subsequent researchers. Autopsies of scorbutic patients by Barlow and Möller revealed subperiosteal hematomas in the subspinous and infra spinous fossae of the scapula, on the ecto and endocranial surfaces of the parietal bones, the orbital roofs, and in the ethmoid (Barlow, 1883, 1894). Furthermore, Barlow noted “thickening” and “swelling” of the zygomatic region, maxillae, mandible, occipital, and parietals in living patients which he attributed to probable scorbutic subperiosteal hemorrhage (Barlow, 1883, p. 168–169).

Unlike subperiosteal new bone, direct clinical evidence for abnormal cortical porosity in scorbutic patients is exceedingly thin (cf. Park, Guild, Jackson, & Bond, 1935), although Barlow noted clustered porosity under a subdural hematoma in a scorbutic child (Barlow, 1883, p. 182). This is probably because this abnormal porosity is not radiographically visible and is not observable in living individuals. However, as discussed above, the relationship between scorbutic hemorrhage and abnormal cortical porosity is logical when the pathophysiology of the disease is considered.

## 6 CRITIQUES OF THE ORTNER CRITERIA

Several of the features noted by Ortner and colleagues, specifically the subperiosteal new bone of the orbital roof and cortical porosity of the greater wing of the sphenoid, as well as the ectocranial parietal, have been noted in documented cases of scurvy in an anatomical collection housed at the Royal College of Surgeons (Stark, 2014). However, the subperiosteal new bone in these anatomical examples is far more severe in expression than the lesions noted by Ortner and colleagues, leading some to critique the use of these features in the diagnosis of scurvy in undocumented skeletal remains (Melikian & Waldron, 2003; Waldron, 2009). However, as Stark (2014) has argued, these anatomical examples likely represent the extreme end of the scorbutic spectrum. The nature and location of the lesion is, in this instance, likely more important than the degree of expression. Additionally, the subtler expression of osseous changes makes identification of scurvy challenging in many cases, as the porotic and subperiosteal nature of the Ortner suite of lesions has considerable overlap with normal infant growth (Snoddy et al., 2017). Because of this, there is a danger that the Ortner criteria may lend itself to confirmation bias, a tendency to interpret new data in support of pre‐existing expectations, especially in analysis of very young individuals who are undergoing rapid appositional and endochondral growth. In other words, subtle changes associated with normal osseous development may be recorded as pathological if the analyst is “looking” for disease. This potential problem has led some researchers to argue that the supposed hemorrhagic lesions described by Ortner and others, while theoretically plausible, should be avoided and that clinically described signs should be considered the only diagnostic features of scurvy in human remains (Melikian & Waldron, 2003). However, we would argue that the rapid clinical resolution of many of the radiographic features of juvenile scurvy following treatment means that most of these lesions would only be present in children who were actively scorbutic at the time of their death. The observation of radiographic features is further complicated by issues with preservation and completeness in archaeological human remains. Unfused epiphyses, for example, are frequently lost and metaphyses are often damaged in the post depositional environment. If these regions are unobservable a differential diagnosis considering clinical radiographic features is impossible.

Stark (2014) attempted to overcome some of these issues by creating a framework for the study of juvenile scurvy in the past by integrating clinical radiographic features with macroscopic osseous pathology. This approach has the potential to critically evaluate the diagnostic robusticity of the hemorrhagic features described by Ortner & colleagues. Stark recommended this framework be applied to collections of juveniles who exhibit macroscopic porous and proliferative lesions. However, its application is not universally practical considering problems with preservation and completeness. Although interpretative caution is needed in order to avoid over diagnosis in paleopathology, the Ortner suite and other proposed hemorrhagic lesions (e.g., new bone around the foramen rotundum; Geber & Murphy, 2012) are logical from an anatomical and pathophysiological standpoint and their continued use in paleopathological research is tenable. However, as discussed in section five, the diagnostic weight attributed to these lesions should be explicitly justified and continually reassessed in paleopathological work.

## 7 ADULT SCURVY: A DIAGNOSTIC CONUNDRUM

Unlike juveniles, the clinical radiographic features of adult scurvy are non‐specific and limited to generalized osteopenia, vertebral collapse, and other signs associated with low bone mineral density, all of which may also occur in adult vitamin D deficiency osteomalacia and age‐related osteoporosis (Hirschmann & Raugi, 1999; Jaffe, 1972 ; Joffe, 1961). Subperiosteal new bone on the shafts of long bones, as well as evidence of hemarthrosis, has been radiographically observed in adults with this disease, but these features also occur in a number of other pathological conditions, including trauma and infectious disease (Jaffe, 1972; Joffe, 1961). As chronic hemorrhage occurs in both scorbutic adults and children, the macroscopic lesions reported by Ortner and others can theoretically be applied to adult individuals and well as subadults (Crandall & Klaus, 2014; e.g., Buckley et al., 2014; Geber & Murphy, 2012). Jaffe (1972) reports that autopsy findings of adults with scurvy include bloody discoloration of muscle bellies and attachments, which lends clinical credibility to this argument (Jaffe, 1972, p. 454). However, the osseous response to hemorrhage is expected to be milder in expression in individuals who have ceased active appositional growth (Crandall & Klaus, 2014). This makes the identification of scurvy in adult skeletal remains from macroscopic lesions difficult and, as with other metabolic bone diseases, juveniles are expected to comprise the largest cohort within a skeletal assemblage who exhibit clear evidence of the disease (see Brickley & Ives, 2008).

The bioarchaeological literature on adult scurvy is comparatively small. Most of the features employed in identification of this condition are nonspecific and include periostitis on the lower limbs, ossified hematomas (suggestive of healed or healing scurvy), periodontal disease (alveolar resorption and porosity), and antemortem tooth‐loss (Crist & Sorg, 2014; Maat, 2004; Van der Merwe et al., 2010). However, Maat (2004) and Van der Merwe et al. (2010) have successfully employed histological and biomolecular methods to confirm the nature of ambiguous skeletal lesions in adult individuals. Maat (2004) used immunoenzymatic staining to confirm the presence of denatured hemoglobin on the tips of dental roots and darkly stained joint surfaces, indicating the presence of alveolar hemorrhage and hemarthroses, respectively. This approach may allow the identification of active scurvy (i.e., prior to the resumption of osteoid formation and formation of subperiosteal new bone), however, it requires excellent preservation (the remains analyzed by Maat were frozen) and the monetary and laboratory resources for biomolecular analysis which are not realistic considerations for all paleopathologists. Likewise, histological analysis of lesions can confirm the presence of subperiosteal new bone and aid in the identification of scurvy in adults with milder lesion presentation (Maat, 2004; Van der Merwe et al., 2010), but the destructive sampling that this requires is not always possible.

Although the other dry‐bone features (osteopenia, etc.) of scurvy in adults may be ambiguous, macroscopic lesions at vascular and entheseal sites consistent with scorbutic hemorrhage have been identified in adult individuals (Buckley et al., 2014; Brickley et al., 2016; Crist & Sorg, 2014; Geber & Murphy, 2012; Snoddy et al., 2017). Furthermore, the pattern of bloody entheseal staining observed in frozen individuals by Maat and colleagues could reasonably be expected to be related to abnormal cortical porosity and/or subperiosteal lesions at these sites in adults suffering from chronic or cyclic deficiency. Because of this, we argue that the macroscopic lesions associated with hemorrhage outlined in the framework below may be applied to adults as well as juveniles. However, we acknowledge that many adult cases are likely to be missed if this is the only diagnostic approach taken and that the histological and biomolecular approaches employed by Maat and others merits further exploration (Koon 2012 in Armelagos et al., 2014; de Boer, Van der Merwe, & Maat, 2013; Maat, 2004; Van der Merwe et al., 2010).

## 8 PATHOGNONOMIC, DIAGNOSTIC, OR SUGGESTIVE? MOVING TOWARD A STANDARDIZED APPROACH

*“If what we are doing in paleopathology is science, we must set out objective criteria for identifying disease in ancient human remains and make our identification procedures explicit”* (Powell and Cook, 2016, p. 42)

As noted, the recent proliferation of research on the paleopathology of scurvy is exciting and has the potential to contribute significantly to our collective understanding of subsistence strategy, human‐environmental interactions, and the biological consequences of social inequality. However, a systematic approach to the diagnosis of this disease in skeletal remains and an exploration of the assignment of the diagnostic weight of individual lesions is needed in order to avoid over‐diagnosis. Although much has been done in terms of methodological advancement over the last two decades (Brickley & Ives, 2006, 2008; Ortner et al., 1999, 2001), the application and diagnostic weight given to osseous features attributed to this disease has not always been uniform. Some researchers consider particular lesions (e.g., porosity of the greater wing of the sphenoid) pathognomonic or strongly diagnostic for this condition (Ortner et al., 1999, 2001; Ortner & Ericksen, 1997), while others do not recognize any strongly diagnostic osseous features (Brickley & Ives, 2008) or simply list all lesions exhibited which have been shown to have any association with the disease (Wrobel, 2014). Despite the advocacy of researchers such as Klaus (2014, 2017) and others (Armelagos et al., 2014; Stark, 2014), there does not currently appear to be a firm consensus within the paleopathological community for best practice in terms of the differential diagnosis of scurvy. Although the clinical literature does not have a strict set of osseous changes required for a diagnosis of scurvy, clinicians have the benefit of laboratory analysis of serum concentrations of ascorbic acid to confirm disease (Stark, 2014). Furthermore, clinicians are unable to visualize many of the osseous changes recorded by paleopathologists since these are generally far too subtle to be manifested radiographically. Because of the different observations available in the clinical and paleopathological literature, we argue that it is imperative that lesions commonly attributed to scurvy in the bioarchaeological literature are assigned diagnostic values according to their association (or lack thereof) with clinical observations, and that lesions with no clinically observed foundation are included only if they have an intuitive relationship with the pathophysiology of this disease.

Brickley and Ives (2008) have created such a weighted diagnostic system for the identification of metabolic bone diseases, including scurvy, in human skeletal remains with various features ascribed “strongly diagnostic”, “diagnostic”, or “suggestive” status with clinical citations. They propose that a diagnosis of scurvy should only be made where multiple diagnostic features are present in a single individual and they do not recognize any strongly diagnostic lesions for this condition. This approach is valuable and could be adapted for use in the differential diagnosis of any number of pathological conditions. However, the diagnostic weight given to various lesions is not explicitly justified by the authors and entire bones (e.g., sphenoid, maxillae), rather than specific osseous sites (e.g., lateral apect of the greater wing of the sphenoid, maxillary palate, etc) are cited (Brickley & Ives, 2008, p. 57). These are important distinctions as a single bone may exhibit multiple osseous lesions of differing diagnostic value. Additionally, this system could be updated with more recent paleopathological research (Buckley et al., 2014; Geber & Murphy, 2012; Moore & Koon, 2017). Although the relative clinical rarity of this disease precludes large observational studies, we argue that a standardized methodological approach to diagnostic values could be created by testing the association of proposed dry‐bone lesions of this disease with scorbutic features for which there is clinical evidence and/or a significant body of paleopathological observations. While case studies and case‐series are a useful starting point for the identification of “new” osseous lesions associated with scurvy (Klaus, 2017; Moore & Koon, 2017; Ortner & Ericksen, 1997), the diagnostic weight of features associated with this disease should be statistically explored whenever sample size permits (Geber & Murphy, 2012). We maintain that features which do not show a statistically significant association with known, previously published diagnostic lesions of scurvy should only be considered suggestive according to the framework proposed by Brickley and Ives (2008). However, statistical means of assigning diagnostic value to “new” lesions requires a baseline of macroscopic features which are accepted to be associated with the disease. We therefore propose that the process of assigning diagnostic weight to features commonly attributed to scurvy in the bioarchaeological literature need not necessarily require statistical analysis (cf. section 6), but the line of reasoning for the diagnostic value assigned to these features must be made explicit by the researcher. For example, a feature assigned “diagnostic” value must not only be intuitive from an anatomical and pathophysiological standpoint, it must also have either the support of clinical observations or a large body of bioarchaeological evidence. A feature assigned “suggestive” value might be one that is anatomically intuitive but which does not have clinical support or has only be observed in a few (<5) bioarchaeological case‐studies or case‐series.

As research on the paleopathology of scurvy continues, diagnostic criteria will naturally evolve and require updating. For instance, features previously noted in small samples may be upgraded from suggestive to diagnostic after additional work with larger samples. An example of how this approach could be applied is provided below.

## 9 AN EXAMPLE OF PRACTICAL APPLICATION: SCURVY IN THE ANCIENT ATACAMA DESERT

### 9.1 Materials and methods

**Figure 3:**
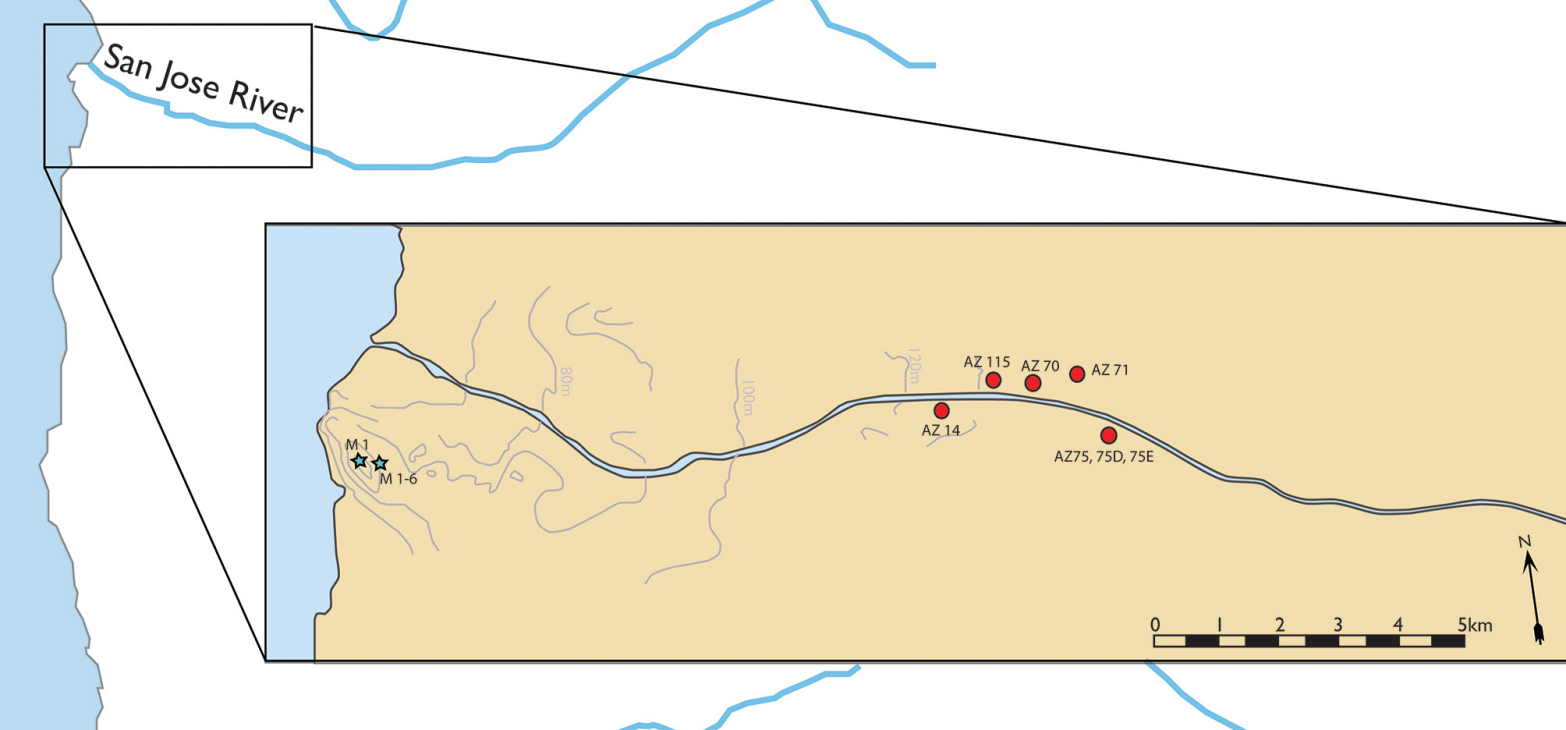
Sites comprising the archaic and formative period cohorts

The Atacama Desert, located in what is now Northern Chile and Southern Peru (18 N‐27S; Figure 3), is the most arid inhabited region in the world (Núñez, Grosjean, & Cartajena, 2002). Rainfall here is extraordinarily scarce (<2 mm a year on average), fertile topsoil formation does not occur, and terrestrial biodiversity is very low (Houston, 2006a, 2006b; Rundel, Dillon, Palma, & Mooney, 1991). Although humans have inhabited this region since the Early Holocene (ca 13,000–9,000 BP), prior to the adoption of contemporary irrigation technology, populations were confined to coastal estuaries and river valleys fed by the seasonal melting of Andean snow (Arriaza, Standen, & Cassman, 2008; Rivera, 2008). The low terrestrial biodiversity and marginal ecology of this region is contrasted by a diverse and abundant marine biomass, fostered by the cold waters of the Pacific Humboldt current (Montecino & Lange, 2009; Tam et al., 2008). This provided the resources necessary for the rise of the Chinchorro Cultural Complex, a sedentary hunter‐gatherer society, during the Archaic Period (9,000–3,500 BP) (Arriaza, 1995; Standen & Arriaza, 2014; Standen, Santoro, Arriaza, & Coleman, 2018). Isotopic analysis of human bone, paleobotanical, and paleofaunal evidence from Archaic Period sites show a heavy reliance on protein‐rich marine resources (Castro, 2014; King et al., 2018b). However, the Formative Period (ca 3,500–1,500 BP) saw population movement from the coast to the interior river valleys and supplementation of marine resources with cultivars such as squash (*Cucurbita spp.*), legumes (*Leguminosae family*), and maize (*Zea mays*) (King et al., 2018b; Núñez & Santoro, 2011; Rivera, 1991). The context of general environmental marginality makes this a unique location for the adoption of agriculture, and indeed the presence of any prehistoric human settlements, a remarkable example of human biosocial adaptation to environmental extremes.

Skeletal analysis of 187 individuals from sites spanning the Archaic (N = 72) and Formative (N = 115) Periods was conducted as part of a larger investigation of the consequences of subsistence transition on human health in this ecologically extreme region. The majority of individuals, including infants and perinates, were well preserved and represented by more than 50% of skeletal elements. Subsequent to age and sex estimation, all surfaces of all elements were examined macroscopically for any abnormalities of bony proliferation and destruction, as well as lesion size, density, and shape. Access to radiographic equipment was limited but wherever possible, analog radiographs were obtained of any individuals exhibiting macroscopic skeletal lesions.

Differential diagnosis of scurvy was conducted using a modified version of Brickley and Ives (2008) system with lesions classified as diagnostic (D) or suggestive (G). This system was modified to include more recent paleopathological literature and additional clinical work (see Snoddy et al., 2017). Two or more D features in a single individual were required for a diagnosis of probable scurvy and at least one D feature and multiple G features were required for diagnosis of possible scurvy.

### 9.2 Results

**Figure 4:**
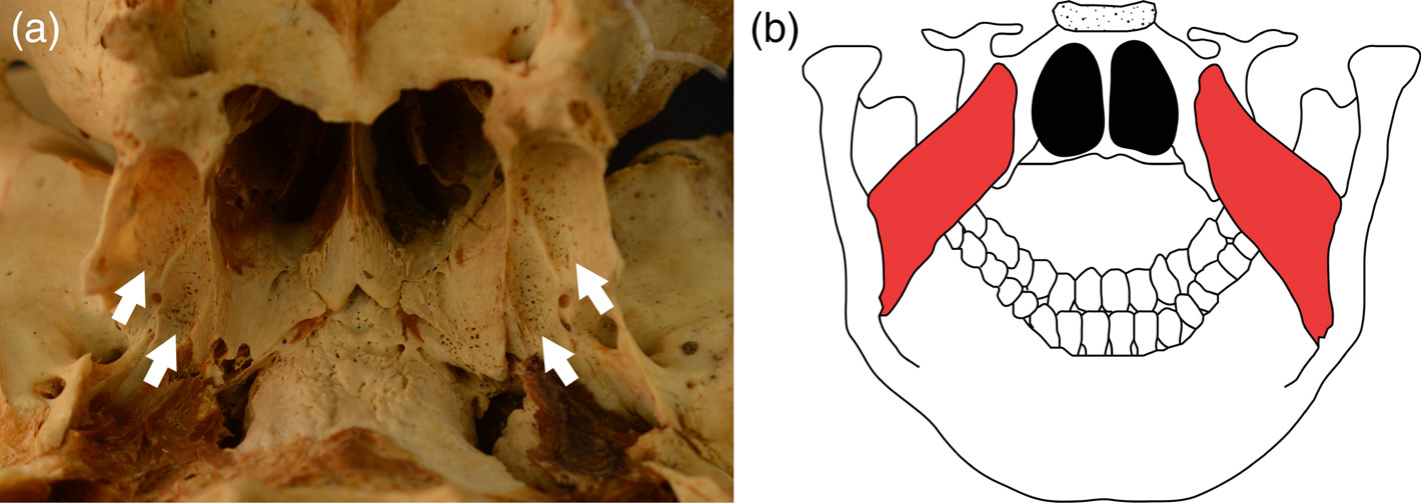
(a) Posterior sphenoid showing porous new bone formation in the pterygoid fossae (white arrows) (b) posterior view of the sphenoid and mandible showing the origin and attachment points of the medial pterygoid muscles (red)

Fifty‐five individuals exhibited at least one diagnostic feature for scurvy with 42 individuals meeting the criteria for probable disease and 13 individuals meeting the criteria for possible disease. Diagnostic and suggestive features of scurvy observed across both temporal cohorts consisted predominantly of subperiosteal new bone formation at vascular and entheseal sites of the cranium and mandible (Table 2). In the course of analysis, two lesions of potential new diagnostic interest were identified. The first of these, abnormal porosity, often accompanied by subperiosteal new bone, within the pterygoid fossae of the posterior sphenoid (Figure 4(a)) was noted in 41.8% (23/55) of individuals who exhibited at least one previously established diagnostic macroscopic feature of scurvy from Brickley and Ives (2008). This feature has previously been noted in two individuals from 17th century San Croix Island (New France) (Crist & Sorg, 2014), and in a case study of a 6‐ to 8‐year‐old child from Colonial Peru (Klaus, 2017). As both Crist and Sorg (2014) and Klaus (2017) have noted, this is an anatomically logical location as this region is the origin of the medial pterygoid muscle, which is involved in the common movements of mastication and facial expression (Standring, 2016; Figure 4(b)). The medial pterygoid is involved in the elevation and contralateral (side‐to‐side) movement of the mandible (Drake, Vogl, & Mitchell, 2015). It is therefore unsurprising that islands of new bone at the origin of the medial pterygoid were found in a substantial proportion of individuals in the Chilean sample who exhibited other diagnostic features of scurvy. The superb preservation in the sample likely facilitated the recognition of this marker, as the pterygoid plates are delicate and unlikely to survive without damage in many archaeological assemblages.

**Table 2:** Frequency of diagnostic and suggestive lesions within the protocol observed in the sample in individuals classified as probable or possible scorbutic cases

	Diagnostic lesions observed							
	Abnormal cortical porosity of the calvarium	SPNB/P in orbital roofs	SPNB/P on interior mandible (rami/coronoid process)	P on the greater wings of the sphenoid	P of maxillary palate	SPNB/P anterior maxillae	SPNB/P posterior maxillae	SPNB/P in the supra/infraspinous fossae of the scapula(e)
**Formative period fn**	3	14	28	6	17	20	14	3
**Archaic period fn**	2	5	6	5	3	4	4	1
**N**	**5**	**19**	**34**	**11**	**20**	**24**	**18**	**4**
	**Suggestive lesions observed**							
	SPNB/P on the anterior/orbital zygoma	Costochrondral rosary	Generalized osteopenia	Islands of endocranial SPNB				
**Formative period fn**	7	2	3	8				
**Archaic period fn**	2	0	3	8				
**N**	**9**	**2**	**6**	**16**				

P  = abnormal cortical porosity (<1 mm across), SPNB = subperiosteal new bone, fn = frequency.

**Figure 5:**
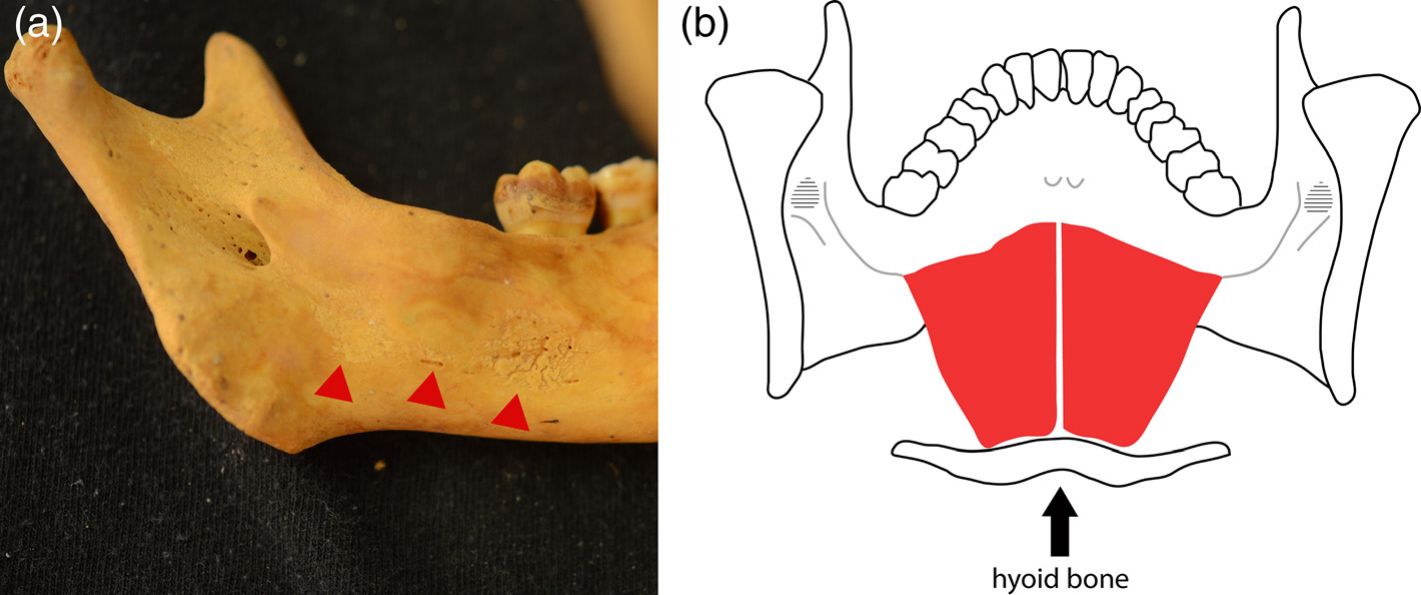
(a) Interior of the mandible of a child (3–4 years, unknown sex) exhibiting islands of active new bone along the mylohyoid line and groove (red arrows) (b) posterior view of the mandible showing the mylohyoid muscle (red) with its origin on the hyoid bone and attachment on the mylohyoid line of the mandible

The second feature of interest was discrete islands of subperiosteal new bone along the mylohyoid line of the mandible (Figure 5(a)) observed in 10.9% (6/55) of individuals who exhibited at least one previously established diagnostic macroscopic feature of scurvy. This lesion has not, to the best of our knowledge, been previously associated with scurvy in the clinical or paleopathological literature. The mylohyoid line is the attachment site of the mylohyoid muscle which originates on the hyoid bone and functions in raising the floor of the oral cavity during mastication and swallowing (Drake et al., 2015; Figure 5(b)). Like the temporalis, masseter, and medial pterygoid, the attachments of the mylohyoid muscle can be expected to be the sites of chronic, low grade hemorrhage in a scorbutic individual as this muscle is essential to small movements of eating, speech, and facial expression.

Exact logistic regression with an odds ratio (OR) output was conducted to test the strength of the association of each of these features with a diagnosis of probable or possible scurvy. A 2 × 2 OR table could theoretically be employed for this approach; however, logistic regression is a more sensitive measure of association when cell counts are under five.

#### 9.2.1 Abnormal cortical porosity in the pterygoid fossa

A significant association was found between this lesion and lesions we considered diagnostic for scurvy according to previously published work (OR 17.879; CI 6.046–64.947; *p* < .001).

#### 9.2.2 Subperiosteal new bone on the mylohyoid line

A significant association was found between this lesion and lesions we considered diagnostic for scurvy according to previously published work (OR 21.399; CI 3.013 ‐ ∞; *p* = .001). In fact, this lesion was *only* exhibited by individuals who also exhibited at least one diagnostic feature for scurvy. However, because of this, the confidence interval is open‐ended and it is impossible to determine the true magnitude of association.

### 9.3 Discussion

We propose on the basis of these data that abnormal cortical porosity in the pterygoid fossae of the sphenoid, with or without abnormal subperiosteal new bone, can be considered a diagnostic feature of scurvy in human skeletal remains. Although new bone on the mylohyoid line has been statistically demonstrated to have an association with other macroscopic features of scurvy, we were unable to characterize the magnitude of this association due to sample size constraints. We therefore propose that this feature be considered suggestive and its diagnostic weight re‐evaluated in future work. The following section incorporates these results into a proposed new weighted diagnostic system for scurvy in human skeletal remains using macroscopic osseous lesions.

## 10 MACROSCOPIC OSSEOUS LESIONS OF ADULT AND JUVENILE SCURVY: AN ANATOMICAL MAP AND WEIGHTED DIAGNOSTIC SYSTEM

“*First, it is key to comparatively and visually map the anatomical distribution of lesions. Then, classification and pattern matching can make equal use of a similar visual framework or rubric to rank potential differential diagnoses to the progressive exclusion of unlikely diagnostic options.*” (Klaus, 2017, p. 101).

The following provides an anatomical guide and weighted diagnostic system for the identification of scurvy from macroscopic skeletal lesions. It consists of a summary of the macroscopic lesions most frequently attributed to scurvy in the paleopathological literature, along with their anatomical associations, and justification for their proposed diagnostic weight. As discussed above, radiographic, histological, and biomolecular methods should be incorporated into the analysis of pathological bone wherever possible. However, we have focused on the macroscopic lesions of scurvy here as these are likely to have the most diagnostic value for paleopathologists working in a field or limited laboratory setting. As with any paleopathological diagnostic system, this methodology is not intended to be exhaustive and will naturally evolve as research on the osseous lesions of scurvy continues to proliferate. Our intention here is to highlight the importance of considering anatomical relationships and the role of soft tissues in lesion formation as well as to illustrate a standardized approach to differential diagnosis.

The weighted diagnostic approach we take here can be summarized as follows: (1) lesions which are anatomically intuitive and either have clinical support or have been associated with scurvy in a large body of bioarchaeological literature are considered *diagnostic* features. (2) Lesions that are anatomically intuitive and have no direct clinical support but have been observed in bioarchaeological case studies or case‐series (*N* < 10 individuals) are considered *suggestive* features. (3) “New” lesions that show a statistically significant association with previously published diagnostic features (e.g., section nine) should be considered *diagnostic* features. As outlined in the example in section nine, an individual must exhibit at least two diagnostic features to be considered a probable disease case and an individual must exhibit at least one diagnostic feature and multiple suggestive features to be considered a possible disease case. The nature of this system means that it is likely that some probable and possible cases of scurvy will be lost if the skeletal remains are incomplete (particularly if the cranium is missing). However, we believe that a conservative approach is necessary in order to avoid overdiagnosis. The complete system is outlined in Table 1. Photographic examples of each feature are provided in Supporting Information Table S1. Each lesion and the justification for its inclusion in this system is described below.

### 10.1 Cranial and Mandibular lesions

#### 10.1.1 Calvarium

**Feature**

Porous subperiosteal new bone on the ectocranial surface of the parietal and/or squamous temporal bones.

**Anatomical association**

**Figure 6:**
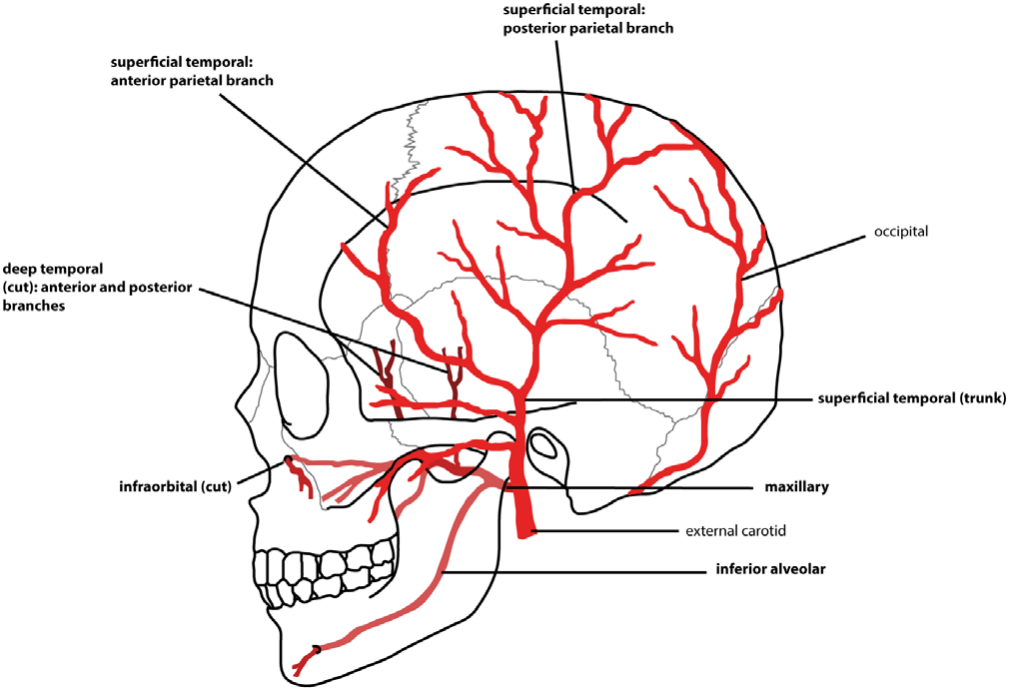
Simplified schematic of the arterial supply of the skull (lateral view). Vessels implicated in osseous scorbutic lesions are in bold

The temporal lines of the lateral parietal are the origin of the temporalis muscle. The squamous portion of the temporal bone underlies the belly and associated vasculature of this muscle (Drake et al., 2015; Figure 6).

**Clinical support**

Ectocranial new bone on the parietal was noted by Sir Thomas Barlow in anatomical material from individuals known to have suffered from scurvy, although these early examples are more extreme than those observed in subsequent paleopathological studies (Stark, 2014).

**Paleopathological research**

Ortner and colleagues have attributed this lesion to scorbutic hemorrhage from vasculature associated with the temporalis, which originates on the lateral aspect of the parietals (Ortner & Ericksen, 1997). Porous subperiosteal new bone has since been described at this location in a large body of paleopathological work (Brickley & Ives, 2006; Brown & Ortner, 2011; Buckley et al., 2014; Geber & Murphy, 2012; Halcrow, Harris, Beavan, & Buckley, 2014; Ortner et al., 1999, 2001).

**Proposed diagnostic strength**

*Diagnostic* if bilateral and subperiosteal nature of new bone can be confirmed. Radiographs or SEM (Brickley & Ives, 2006) should be employed wherever possible to confirm the subperiosteal nature of the new bone.

**Differential Diagnosis**

Trauma, infection, anemia.

**Caveat**

Lesions are expected to be bilateral if attributable to scurvy.

**Feature**

Subperiosteal new bone with vascular impressions and/or fine cortical porosity on the endocranial surface of the vault.

**Anatomical association**

Chronic or acute hemorrhage of the vasculature of the meninges can cause blood clots to form in the subdural space (subdural hematomas) (Yadav, Parihar, Namdev, & Bajaj, 2016).

**Clinical support**

Endocranial porosity underlying a hematoma was noted by Barlow (1883) in an autopsy of a scorbutic individual. Subdural hematomas are a clinically reported complication of scorbutic hemorrhage (Ingalls, 1936).

**Paleopathological research**

This feature has been noted in a number of archaeological individuals who exhibit other features diagnostic of scurvy (Brown & Ortner, 2011; Mays, 2008; Snoddy et al., 2017).

**Proposed diagnostic strength**

Despite its clinical association with scurvy, endocranial new bone and/or porosity have multiple etiologies (Lewis, 2004). As such, we propose that this feature be considered *suggestive.*

**Differential diagnosis**

Any condition that might result in localized inflammation of the meninges, including trauma and infection.

**Feature**

Abnormal cortical porosity and/or subperiosteal new bone in the orbital roofs/ lateral orbit.

**Anatomical association**

Indirect hemorrhage from vessels associated with the extrinsic muscles of the eye might cause blood to pool in this region.

**Clinical support**

Indirect: intraocular hematomas have been noted in cases of active scurvy by Barlow (1883) and Verma et al. (2007).

**Paleopathological research**

This feature has been noted in a large body of paleopathological work (Brickley & Ives, 2006; Klaus, 2014; Ortner et al., 2001; Ortner & Ericksen, 1997).

**Proposed diagnostic strength**: *Diagnostic.*

**Differential diagnosis**: Trauma, anemia.

**Caveat**: Feature is expected to be bilateral if attributable to scurvy. Caution is needed to differentiate cortical porosity from mild cribra orbitalia (trabecular expansion).

#### 10.1.2 Maxillae

**Feature**

Abnormal cortical porosity and/or porous subperiosteal new bone on the anterior surface and/or in the region of the infraorbital foramina.

**Anatomical association**

The infraorbital artery runs through the infraorbital foramen and the buccinator originates on the alveolar process of the anterior maxillae (Drake et al., 2015; Figure 6).

**Clinical support**

None but anatomically intuitive.

**Paleopathologial research**

This feature was first described by Ortner et al. (1999), in 23 individuals who also exhibited abnormal porosity of the greater wing of the sphenoid. It has subsequently been observed in a number of archaeological individuals who exhibit other diagnostic features of scurvy (Brickley & Ives, 2006; Geber & Murphy, 2012; Snoddy et al., 2017).

**Proposed diagnostic strength**

*Diagnostic.*

**Differential diagnosis**

Any condition that might result in localized periosteal inflammation, including trauma and infection.

**Caveat**

Lesions are expected to be bilateral if attributable to scorbutic hemorrhage.

**Feature**

Abnormal cortical porosity and/or porous subperiosteal new bone on the posterior surface, superior to the alveolar margins.

**Anatomical association**

This region is the origin of the superficial head of the medial pterygoid muscle, which is supplied by the medial pterygoid branches of the maxillary artery (Drake et al., 2015).

**Clinical support**

None, but anatomically intuitive.

**Paleopathological research**

This feature was first noted by Ortner and Ericksen (1997). It has subsequently been noted in association with a number of other diagnostic features of scurvy in a large body of paleopathological work (Geber & Murphy, 2012; Klaus, 2014; Ortner et al., 1999, 2001; Snoddy et al., 2017).

**Proposed diagnostic strength**

*Diagnostic.*

**Differential diagnosis**

Porosity associated with alveolar resorption during dental eruption.

**Caveat**

This feature is expected to be bilateral if attributable to scorbutic hemorrhage and care is needed in juveniles to differentiate from normal porosity associated with eruption of the molars.

**Feature**

Abnormal cortical porosity and/or porous subperiosteal new bone on the maxillary palate, outside of the alveolar margins.

**Anatomical association**

The greater palatine artery supplies this region (Drake et al., 2015).

**Clinical support**

None but anatomically intuitive.

**Paleopathological research**

This feature was described by Ortner et al. (1999) in 17 individuals who also exhibited abnormal porosity of the greater wing of the sphenoid. It has subsequently been described in a large body of paleopathological work (Brickley & Ives, 2006; Crist & Sorg, 2014; Geber & Murphy, 2012; Halcrow et al., 2014; Ortner et al., 2001).

**Proposed diagnostic strength**

*Diagnostic.*

**Caveat**

Caution is needed in juveniles due to the normal porosity associated with dental eruption.

#### 10.1.3 Zygomata

**Feature**

Abnormal cortical porosity and/or subperiosteal new bone on the lateral portion of the anterior surface.

**Anatomical association**

**Figure 7:**
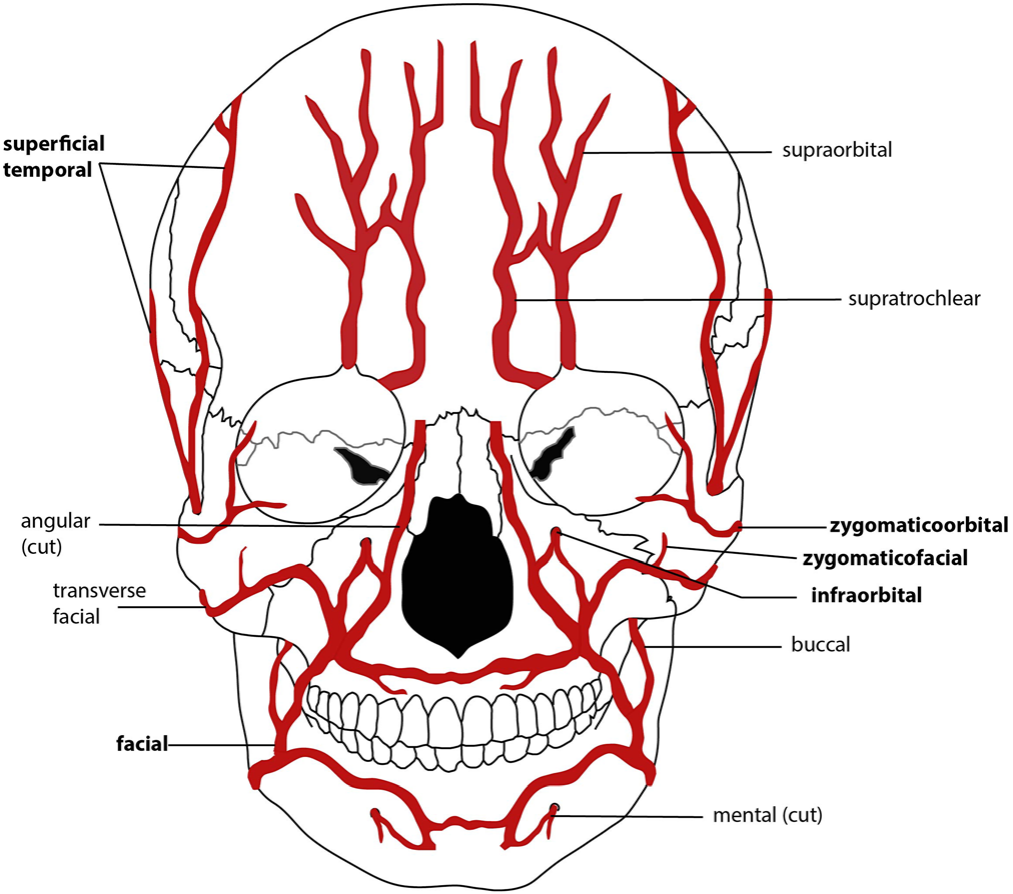
Simplified schematic of the arterial supply of the skull (anterior view). Vessels implicated in osseous scorbutic lesions are in bold

The zygomaticotemporal and zygomaticofacial arteries supply this region and are under indirect stress from orbicularis oculi (Drake et al., 2015; Figure 7).

**Clinical support**

None but anatomically intuitive.

**Paleopathological research**

This lesion has been described in two individuals who exhibited other diagnostic features of scurvy (Pitre, Stark, & Gatto, 2016; Snoddy et al., 2017).

**Proposed diagnostic strength**

*Suggestive.* Direct clinical support and/or demonstration of statistical association with diagnostic features in a larger sample is needed.

**Caveat**

Feature expected to be bilateral if attributable to scorbutic hemorrhage.

**Feature**

Abnormal cortical porosity on the internal/posterior surface.

**Anatomical association**

This region is supplied by the masseteric branches of the maxillary artery and is the origin of the masseter muscle (Drake et al., 2015). Hemorrhage from the orbital and facial branches of the superficial temporal arteries would also result in blood pooling here (Ortner et al., 2001).

**Clinical support**

None but anatomically intuitive.

**Paleopathological research**

This lesion has been described in a handful of paleopathological case studies of scurvy in association with other diagnostic features (Klaus, 2014; Ortner et al., 2001).

**Proposed diagnostic strength**

*Suggestive.* Direct clinical support and/or demonstration of statistical association with diagnostic features in a larger sample is needed.

**Caveat**

This feature expected to be bilateral if attributable to scorbutic hemorrhage.

#### 10.1.4 Sphenoid

**Feature**

Abnormal cortical porosity on the external surface of the greater wing.

**Anatomical association**

This region underlies the belly of the temporalis muscle and is supplied by the anterior deep temporal artery (Drake et al., 2015; Figure 6).

**Clinical support**

This feature has been reported in anatomical material from individuals known to have suffered from scurvy, although these examples are more extreme than has been observed in subsequent paleopathological studies (Stark, 2014). Ortner and colleagues have proposed that these lesions are indirectly caused by masticatory stress from the temporalis muscle on the anterior branch of the deep temporal artery (Ortner & Ericksen, 1997).

**Paleopathological research**

Ortner has argued that this lesion is strongly diagnostic for scurvy and this feature has been described in a large body of paleopathological work collectively comprising scores of individuals (Buckley et al., 2014; Geber & Murphy, 2012; Klaus, 2014; Ortner et al., 2001; Snoddy et al., 2017).

**Proposed diagnostic strength**

*Diagnostic.*

**Feature**

Subperiosteal new bone around the foramen rotundum.

**Anatomical association**

The vasculature associated with the maxillary branch of the trigeminal nerve (V2) passes through here (Drake et al., 2015).

**Clinical support**

None but anatomically intuitive.

**Paleopathological research**

This feature has been reported in 18% (78/412) of juvenile individuals exhibiting lesions within the Ortner suite in a large archaeological assemblage from 19th Century Ireland (N = 970; Geber & Murphy, 2012). This study demonstrated a statistically significant association between this lesion (Geber & Murphy, 2012) and previously published features the authors considered diagnostic (Brickley & Ives, 2008; Brown & Ortner, 2011; Ortner et al., 1999). It has also been observed in several smaller case series and case studies (Snoddy et al., 2017).

**Proposed diagnostic strength**

Tentatively *diagnostic* but association with scurvy should be statistically explored in other samples.

**Differential diagnosis**

Appositional growth (juveniles).

**Feature**

Abnormal cortical porosity on the lesser wings.

**Anatomical association**

These structures do not come into direct contact with muscles or vasculature, but the anterior cerebral artery, anterior communicating artery of the brain, and the orbital branch of the middle meningeal artery are transmitted through this region (Drake et al., 2015).

**Clinical support**

None, but anatomically intuitive.

**Paleopathological research**

This lesion was described in a single infant in a case‐series by Brickley and Ives (2006).

**Proposed diagnostic strength**

*Suggestive.* Direct clinical support and/or demonstration of statistical association with diagnostic features in a larger sample is needed.

**Differential diagnosis**

Normal variation, appositional growth.

**Feature**

Abnormal cortical porosity and/or subperiosteal new bone in the pterygoid fossae.

**Anatomical association**

Lesion location is the origin of the medial pterygoid muscle (Drake et al., 2015; Figure 4(a)(b)).

**Clinical support**

None but anatomically intuitive.

**Paleopathological research**

See section nine.

**Proposed diagnostic strength**

*Diagnostic*; see section nine.

**Differential diagnosis**

Appositional growth (juveniles).

**Caveat**

Feature is expected to be bilateral if attributable to scorbutic hemorrhage.

#### 10.1.5 Occipital

**Feature**

Abnormal cortical porosity on the inferior surface of pars basilaris (**juveniles only**).

**Anatomical association**

This lesion is found in the region of the insertion of the longus capitus muscle (Drake et al., 2015).

**Clinical support**

None, although neck pain has been reported in scorbutic patients (Olmedo et al., 2006).

**Paleopathological research**

This lesion has been reported in a small case‐series (*N* = 9/10) of individuals who exhibited other lesions the authors considered diagnostic for scurvy (Moore & Koon, 2017).

**Proposed diagnostic strength**

*Suggestive.* Direct clinical support and/or demonstration of statistical association with diagnostic features in a larger sample is needed. The etiology of this feature has also been disputed by González et al. (2018).

**Differential diagnosis**

Normal growth.

#### 10.1.6 Mandible

**Feature**

Abnormal cortical porosity and/or subperiosteal new bone on the interior surface of the rami/ coronoid process.

**Anatomical association**

The temporalis muscle inserts on the coronoid process and the inferior alveolar artery passes through the mandibular foramen on the medial aspect of the ramus (Drake et al., 2015).

**Clinical support**

None but anatomically intuitive.

**Paleopathological research**

This lesion was found in three individuals who also exhibited abnormal porosity of the greater wing of the sphenoid (Ortner et al., 1999) and has subsequently been described in association with other scorbutic lesions in a large body of paleopathological work (Geber & Murphy, 2012; Klaus, 2014; Schattmann, Bertrand, Vatteoni, & Brickley, 2016; Snoddy et al., 2017).

**Proposed diagnostic strength**

*Diagnostic.*

**Caveat**

Feature is expected to be present bilaterally if attributable to scorbutic hemorrhage.

**Feature**

Subperiosteal new bone along the mylohyoid line on the interior surface of the body.

**Anatomical association**

This region is the origin of the mylohyoid muscle, which is supplied by the inferior alveolar artery (Drake et al., 2015; Figure 5(a)(b)).

**Clinical support**

None but anatomically intuitive (see section nine).

**Paleopathological research**

Observed in four individuals who exhibited multiple diagnostic lesions for scurvy after Brickley and Ives (2008).

**Proposed diagnostic strength**

*Suggestive.* Direct clinical support and/or demonstration of statistically significant association with diagnostic features in a larger sample is needed.

### 10.2 Axial skeleton (post cranial)

#### 10.2.1 Scapulae

**Feature**

**Figure 8:**
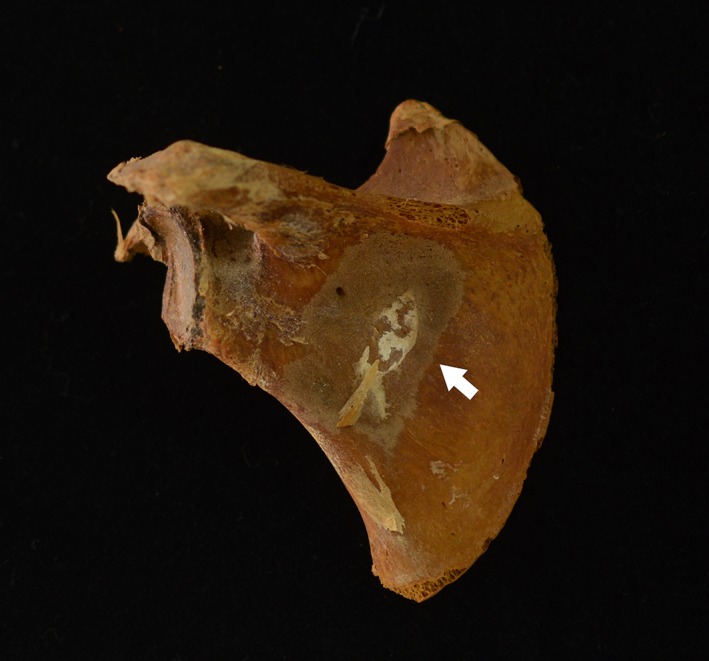
Posterior view of the left scapula of a child with probable scurvy exhibiting an island of subperiosteal new bone in the infraspinous fossa (white arrow)

Abnormal cortical porosity and/or subperiosteal new bone in the supra and/or infraspinous fossae (Figure 8).

**Anatomical association**

The supraspinatus muscle originates within the supraspinous fossa and scorbutic hemorrhage of the associated suprascapular artery could cause extravasated blood to pool here (Drake et al., 2015). Likewise, the infraspinatus and teres minor muscles both originate in the infraspinous fossa and scorbutic hemorrhage of the circumflex branch of the subscapular artery might be caused by lateral rotation of the arm (Drake et al., 2015).

**Clinical support**

Indirect; Barlow (1883) noted subperiosteal hematomas in these regions in autopsies of scorbutic children.

**Paleopathological research**

Ortner et al. (2001) noted these features in several individuals who also exhibited abnormal porosity of the greater wing of the sphenoid. These lesions have subsequently been described in several other paleopathological studies in association with other scorbutic lesions (Brickley & Ives, 2006; Brown & Ortner, 2011; Buckley et al., 2014; Geber & Murphy, 2012; Klaus, 2014).

**Proposed diagnostic strength**

*Diagnostic.*

**Differential diagnosis**

Trauma, appositional growth.

#### 10.2.2 Ribs

**Feature**

Enlargement of the costochrondral junction (**juveniles only**).

**Clinical support**

This feature is part of the radiographic suite of lesions seen in pediatric scurvy and is caused by defective collagen formation at the sternal margins of the ribs (Jaffe, 1972). However, it is more strongly associated with rickets (Adams, 2011).

**Paleopathological research**

Buckley et al. (2014) describe this feature in a perinate from prehistoric Vanuatu, and Schattmann et al. (2016) have described it in several potential cases of comorbid scurvy and rickets.

**Proposed diagnostic strength**

Despite its clinically demonstrated association with scurvy, we consider this feature *suggestive* as it is more frequently found in association with rickets and the paleopathological evidence for its association with scurvy is thin.

**Differential diagnosis**

Rickets.

**Feature**

Subperiosteal new bone on the anterior/lateral surfaces of the shafts.

**Anatomical association**

This region underlies the pectoralis major (anterior surface), pectoralis minor (antero‐lateral), and serratus anterior (antero‐lateral) muscles (Drake et al., 2015).

**Clinical support**

Limited; Barlow (1883) has described the presence of subperiosteal hematomas on the shafts of ribs in autopsied scorbutic children.

**Paleopathological research**

This feature has been described in two perinates exhibiting other lesions diagnostic for scurvy (Buckley et al., 2014; Snoddy et al., 2017).

**Proposed diagnostic strength**

*Suggestive.* This feature is associated with a number of other pathological conditions. Direct clinical support and/or demonstration of statistically significant association with diagnostic features in a larger sample is needed.

**Differential diagnosis**

normal appositional growth, trauma, infection.

**Caveat**

Feature is expected to be bilateral and widespread.

#### 10.2.3 Vertebrae

**Feature**

Biconcavity and osteopenia of bodies (adults only).

**Clinical support**

This feature has been clinically associated with adult scurvy (Joffe, 1961). However, it is also found in osteomalacia and age‐related osteoporosis (Jaffe, 1972).

**Paleopathological research**

We have been unable to find published paleopathological research ascribing this feature to scurvy.

**Proposed diagnostic strength**

Despite its clinically demonstrated association with scurvy, we consider this feature *suggestive* due to its commonality across several metabolic bone diseases.

**Differential diagnosis**

Osteomalacia, age‐related osteoporosis, trauma.

**Caveat**

Biconcavity due to metabolic bone disease rather than trauma is expected to be widespread throughout the vertebral column (Jaffe, 1972; Resnick, 1995).

#### 10.2.4 Ilia

**Feature**

Abnormal cortical porosity and/or subperiosteal new bone, particularly on the visceral surface. Subperiosteal new bone may contain vascular impressions.

**Anatomical associations**

This region underlies the belly of the iliacus muscle, which is supplied by the internal iliac artery (Drake et al., 2015).

**Clinical support**

Indirect; Barlow (1883) noted subperiosteal hemorrhage on an ilium of an autopsied scorbutic child and subperiosteal fluid around the ilium and ischium has been visualized by MRI in a clinical case‐report (Gongidi, Johnson, & Dinan, 2013).

**Paleopathological research**

This feature was first noted in a case‐study by Brown and Ortner (2011) and has subsequently been described in several individuals by Geber and Murphy (2012).

**Proposed diagnostic strength**

*Suggestive.* Additional clinical support and/or demonstration of statistical association with diagnostic features in a larger sample is needed.

**Differential diagnosis**: Infection, trauma.

### 10.3 Appendicular skeleton

#### 10.3.1 General changes

**Feature**

Widespread, porous, subperiosteal new bone on the diaphyses/metaphyses of long bones (adults and juveniles).

**Anatomical associations**

Multiple. The diaphysis/metaphyses of the appendicular long bones underlie the bellies of numerous muscles with a variety of vascular innervations. Widespread subperiosteal bleeding in the appendicular skeleton can occur due to minor soft tissue trauma in scorbutic individuals (Besbes et al., 2010; Jaffe, 1972; Joffe, 1961).

**Clinical support**

This feature has been clinically observed following the reintroduction of vitamin C and resumption of osteoid formation (Noordin et al., 2012; Riepe et al., 2001).

**Paleopathological research**

Ortner et al. (2001) noted this feature in several individuals who also exhibited abnormal porosity of the greater wing of the sphenoid. It has subsequently been noted in a large body of paleopathological work in association with other osseous features of scurvy (Brown & Ortner, 2011; Buckley et al., 2014; Geber & Murphy, 2012; Klaus, 2014; Snoddy et al., 2017; Van der Merwe et al., 2010).

**Proposed diagnostic strength**

*Diagnostic* if found in association with cranial lesions.

**Differential diagnosis**

Trauma, infection.

**Caveat**

Expected to be present bilaterally if attributable to scorbutic hemorrhage. Care is needed with interpretation in juveniles as this is also a feature of appositional growth.

**Feature**

Discrete ossified hematomas.

**Anatomical association**

Multiple (see above).

**Clinical support**

As above, this feature has been clinically observed following the re‐introduction of vitamin C and resumption of osteoid formation (Brailsford, 1953).

**Paleopathological research**

The paleopathological observations of this feature in association with scurvy come from adult individuals (Buckley et al., 2014; Maat, 2004; Van der Merwe et al., 2010).

**Proposed diagnostic strength**

*Suggestive* if bilateral, non‐diagnostic if unilateral.

**Differential diagnosis**

Trauma, osteogenesis imperfecta, hemophilia.

**Feature**

Metaphyseal cupping/ flaring (**juveniles**).

**Clinical support**

Limited; a case study by Sprague (1976) described metaphyseal cupping in a scorbutic individual several years following recovery. This lesion occurred due to epiphyseal slipping from the residual effects of scurvy on the provisional zone of calcification of the growth plate. This feature has a stronger clinical association with rickets (Adams, 2011; Jaffe, 1972).

**Paleopathological research**

Schattmann and colleagues (2016) have described metaphyseal cupping in possible comorbid cases of scurvy and rickets.

**Proposed diagnostic strength**

*Suggestive.*

**Differential siagnosis**

Rickets.

#### 10.3.2 Femora

**Feature**

Subperiosteal new bone on the proximal and medial diaphysis, medial to the linea aspera.

**Anatomical association**

This region is the insertion of the adductor longus muscle, which is supplied by the profundus femoris artery (Drake et al., 2015).

**Clinical support**

None but anatomically intuitive.

**Paleopathological research**

Buckley et al. (2014) in several individuals who exhibited other osseous features of scurvy.

**Proposed diagnostic strength**

*Suggestive.* Direct clinical support and/or demonstration of statistical association with diagnostic features in a larger sample is needed.

**Differential diagnosis**

Activity related entheseal changes, enthesopathies.

## 11 SUMMARY AND CONCLUSIONS

The recent proliferation of research on scurvy in ancient human remains is promising and carries implications for how we study subsistence, resource allocation, and human‐environmental interactions in the past. However, the diagnostic criteria for scurvy employed by paleopathologists has not always been uniform. This article has provided a literature synthesis of the previous body of work on the identification of scurvy in archaeological human remains. Drawing from this foundation, we have proposed a standardized approach to differential diagnosis that assigns diagnostic value based on the strength of association between macroscopic osseous lesions, clinically observed features, and anatomical relationships. Using a well‐preserved sample from the ancient Atacama Desert, we have also demonstrated an approach for assigning diagnostic weight to newly recognized lesions of this disease.

We encourage debate on this subject and consider this work a starting point for a larger academic conversation that will ultimately refine diagnostic protocol and increase the rigor of this system. Our intention here was to highlight the need for a standardized approach which considers the pathophysiology of the disease and soft‐tissue anatomy. It is our hope that other researchers will carry this approach forward and continue to advance the study of this condition in past‐populations.

## Supporting information


**Supplementary Table S1**: Visual examples in dry bone of diagnostic and suggestive features within the proposed weighted diagnostic system. These images are intended as guidelines only; the severity of lesion expression may vary between affected individuals.
