# Supplementary material for: Macroscopic features of scurvy in human skeletal remains: A literature synthesis and diagnostic guide
Source: Am J Phys Anthropol. 2018 Oct 9;167(4):876–95. doi: 10.1002/ajpa.23699 (PMC6282809; doi:10.1002/ajpa.23699)
Supplement: Supplementary file 1 — Supplementary Table S1: Visual examples in dry bone of diagnostic and suggestive features within the proposed weighted diagnostic system. These images are intended as guidelines only; the severity of lesion expression may vary between affected individuals. [file AJPA-167-876-s001.pdf]

Lesion Location	Lesion Type	Diagnostic Strength	Example	Image notes
Ectocranial Parietal/Squamous Temporal	Abnormal cortical porosity, SPNB	Diagnostic	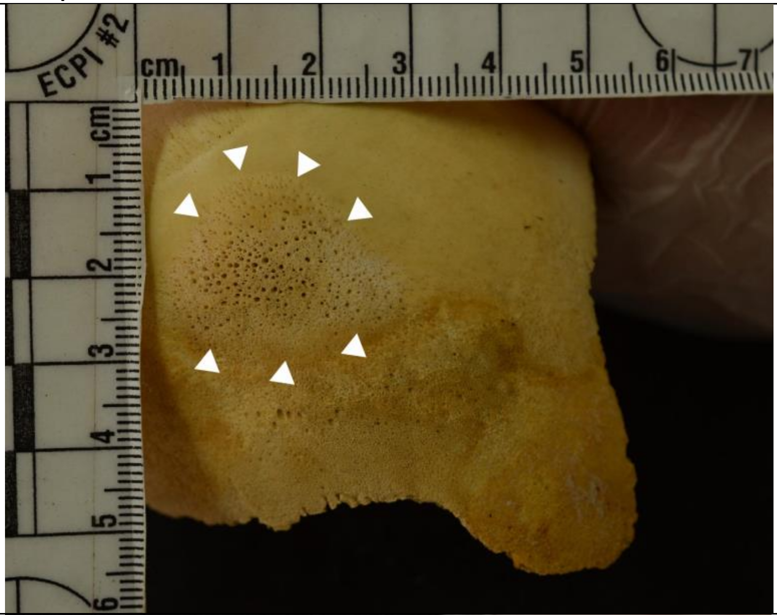	Right parietal of an infant (3-6 months) exhibiting an island of porous subperiosteal new bone on the ectocranial surface. Individual is AZ 71 T168 (Azapa Valley, Formative Period).
Endocranial Calvaria	Abnormal cortical porosity, discrete islands of SPNB	Suggestive	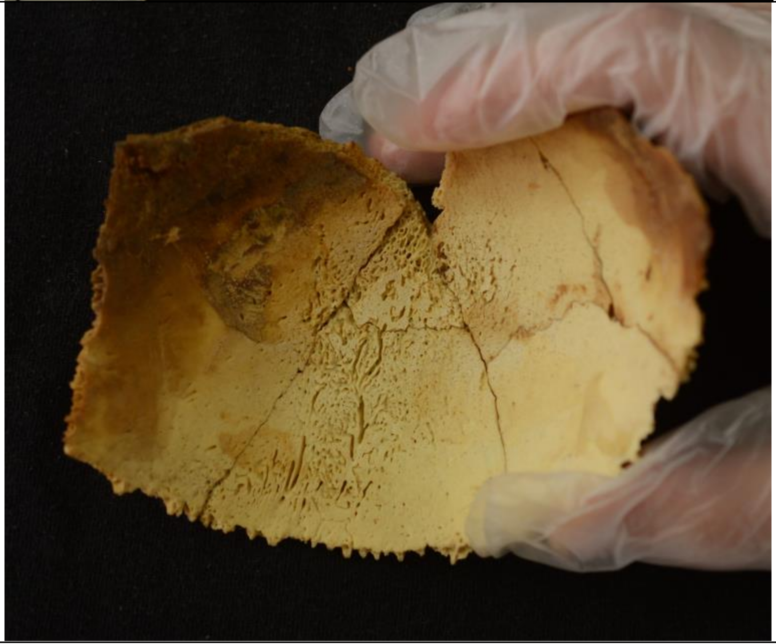	Parietal of a child (~18 months) exhibiting heavy apposition of subperiosteal new bone with vascular impressions on the endocranial surface. Individual is AZ 115 T8a (Azapa Valley, Formative Period).

Sphenoid: greater wing	Abnormal cortical porosity, SPNB	Diagnostic	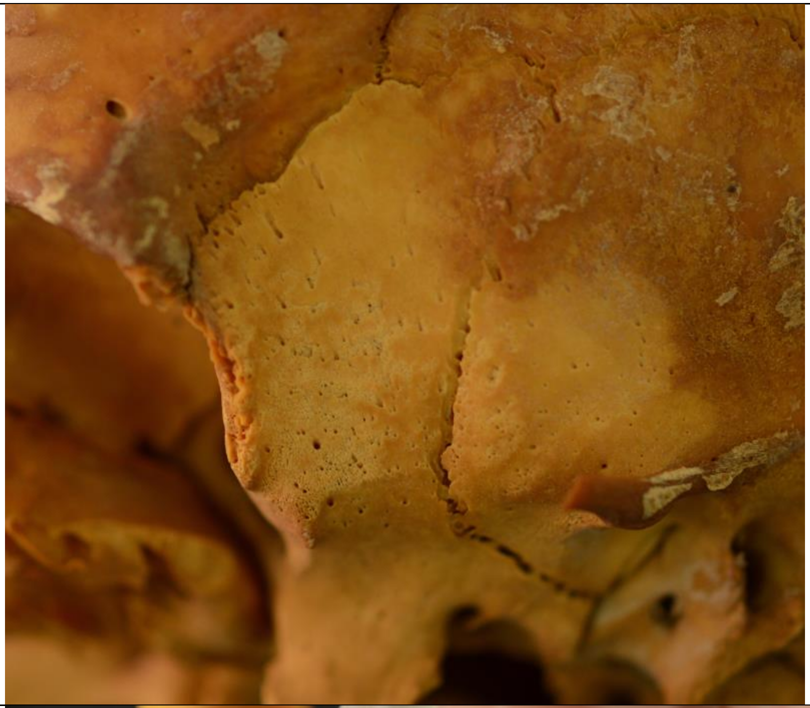	External surface of the left greater wing of the sphenoid of a child (~3 years) exhibiting heavy cortical porosity with clear vascular channels. Individual is AZ 75D T16 (Azapa Valley, Formative Period).
Sphenoid: foramen rotundum	SPNB	Diagnostic	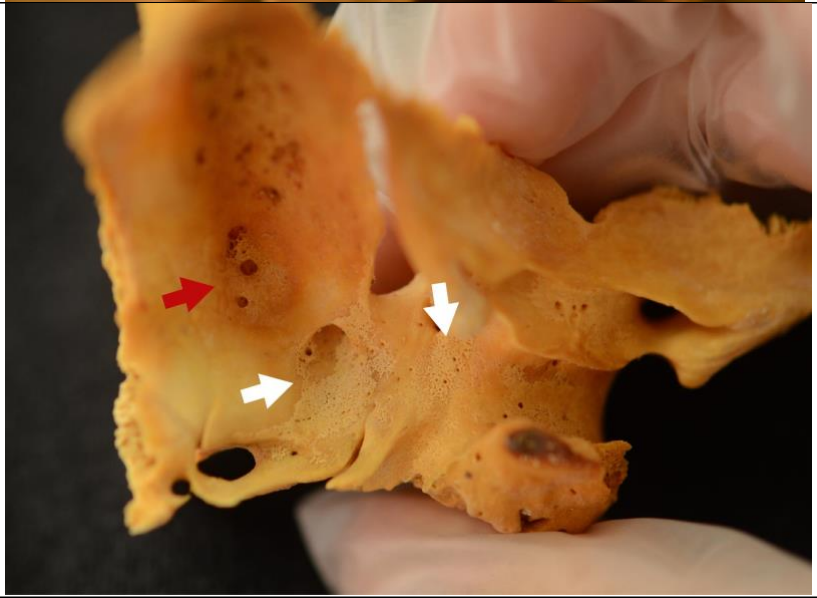	Sphenoid of a child (~18 months) showing islands of subperiosteal new bone around the foramen rotundum and body (white arrows) and well as porosity on the internal surface of the greater wing (red arrow). Individual is AZ 75 TBd (Azapa Valley, Formative Period).
Sphenoid: lesser wing	Abnormal cortical porosity	Suggestive	See Brickley and Ives, 2006: Figure 8	NA

Sphenoid: pteryoid fossae, plates	Abnormal cortical porosity, SPNB	Diagnostic	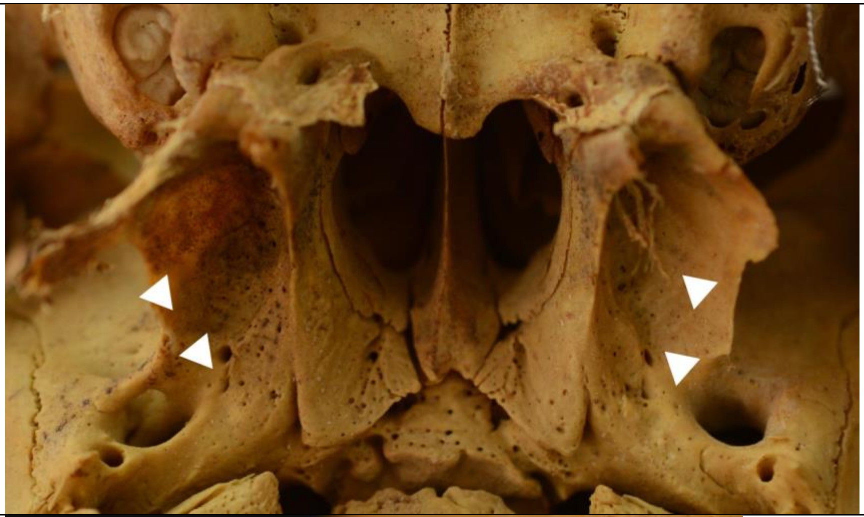	Posterior sphenoid of a child (~6 years) showing heavy cortical porosity in the pterygoid fossae and on the plates (white arrows). Porosity is heavier on the left side. Individual is AZ 75 T81 (Azapa Valley, Formative Period).
Frontal: orbital roof	Abnormal cortical porosity, SPNB	Diagnostic	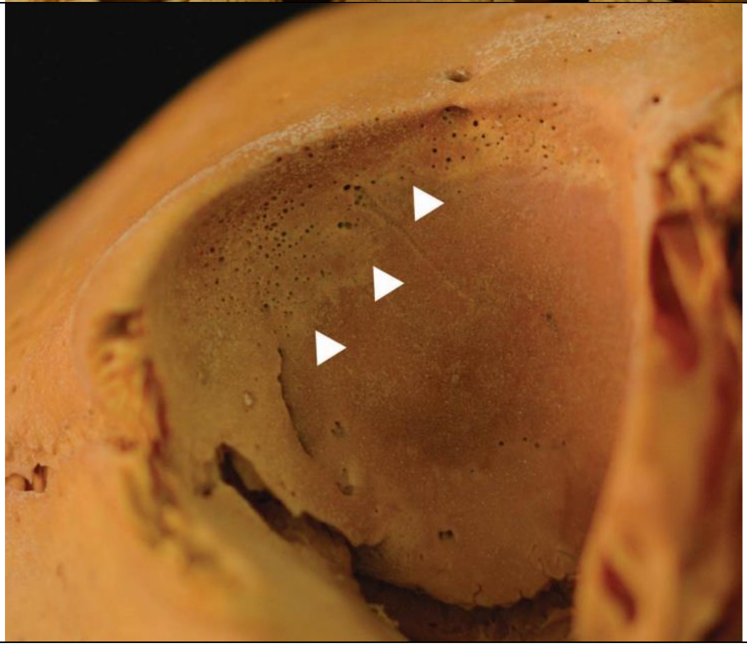	Right orbital roof of a child (~3 years) exhibiting a discrete island of porous subperiosteal new bone along the antero-lateral margins. Individual is AZ 75D T16 (Azapa Valley, Formative Period).
Zygomatic: lateral aspect	Abnormal cortical porosity, SPNB	Suggestive	See Snoddy et al., 2017: Figure 2a	NA

Zygomatic: internal (posterior) aspect	Abnormal cortical porosity	Suggestive	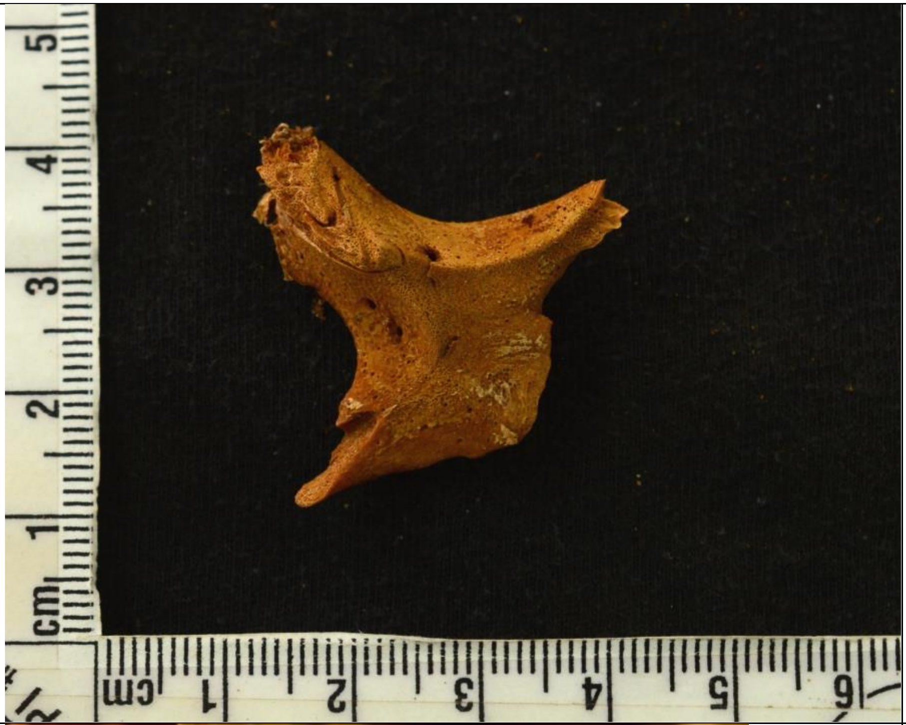	Posterior aspect of the left zygomatic of a child (~1 year) exhibiting a diffuse layer of active subperiosteal new bone covering most of the surface. Individual is AZ 75 T9c (Azapa Valley, Formative Period).
Maxillae: anterior surface/ infraorbital foramina	Abnormal cortical porosity, SPNB	Diagnostic	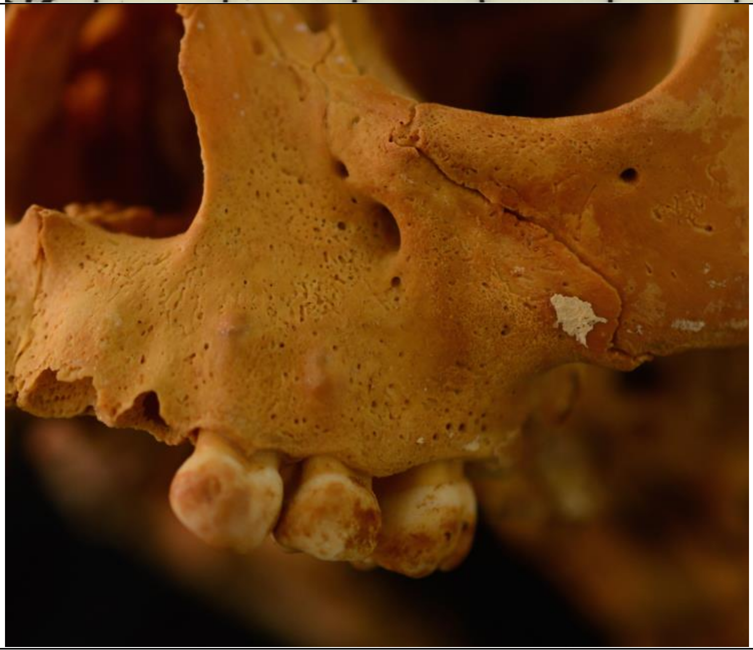	Left maxilla of a child (2-3 years) exhibiting diffuse apposition of active subperiosteal new bone with vascular impressions on the anterior surface. Individual is AZ 75D T22 (Azapa Valley, Formative Period).

Maxillae: posterior surface	Abnormal cortical porosity, SPNB	Diagnostic	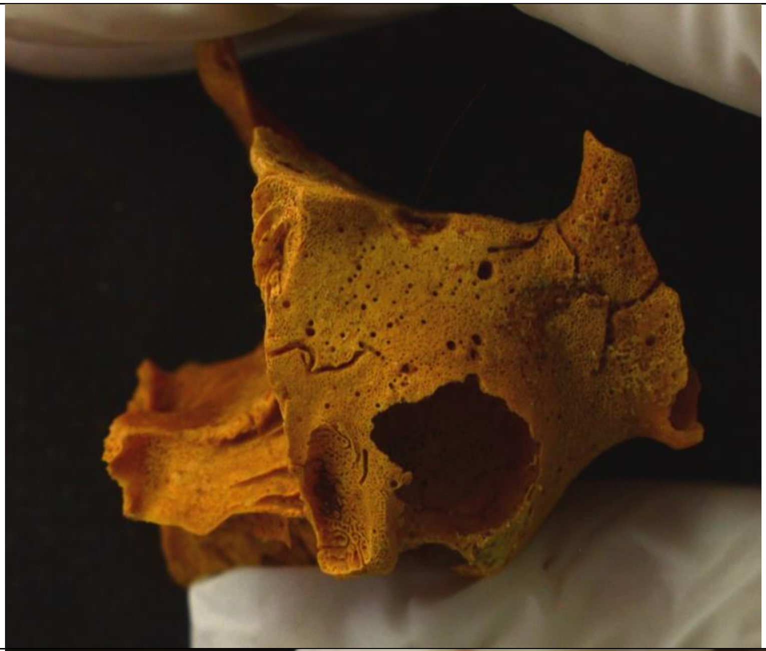	Posterior surface of the right maxilla of a child (9 months – 1 year) exhibiting heavy apposition of porous subperiosteal new bone with vascular impressions. Individual is AZ 75 T12c (Azapa Valley, Formative Period).
Maxillae: palatal surface	Abnormal cortical porosity, SPNB	Diagnostic	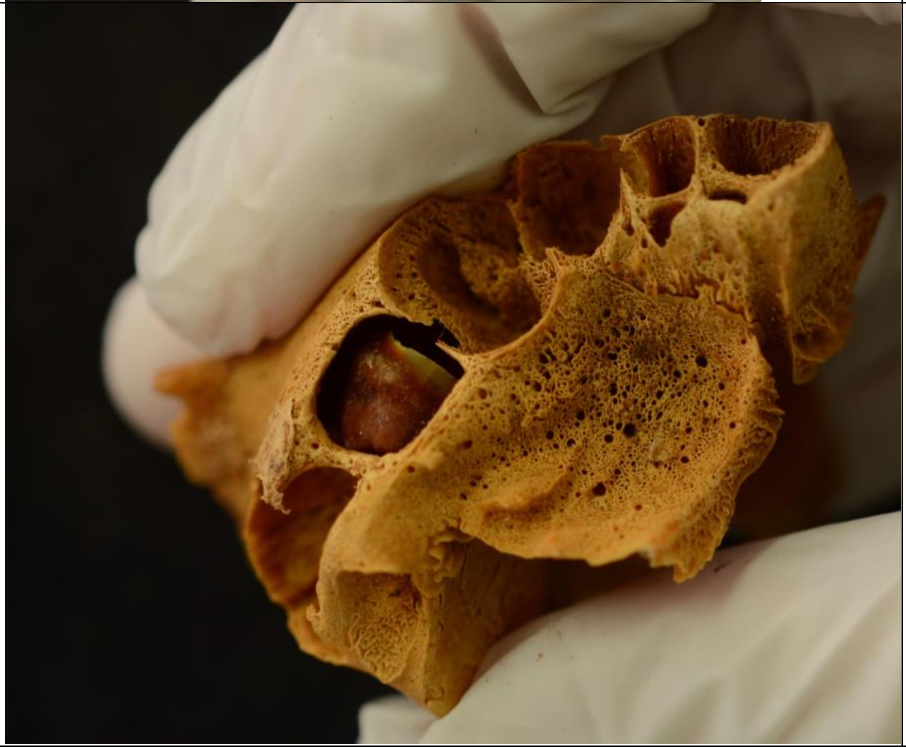	Palatal surface of the right maxilla of a child (9 months-1 year) exhibiting abnormal porosity and heavy apposition of subperiosteal new bone. Individual is AZ 75 T12c (Azapa Valley, Formative Period).

Mandible: medial surface/coronoid process	Abnormal cortical porosity, SPNB	Diagnostic	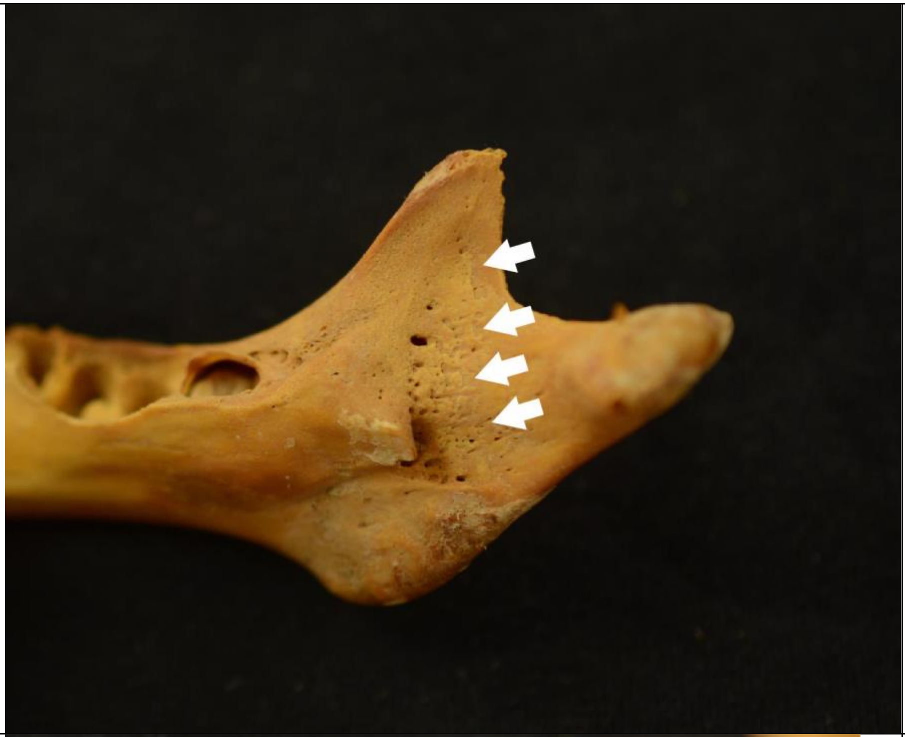	Medial aspect of the right half of the mandible of a child (~18 months) exhibiting active subperiosteal new bone around the mandibular foramen and extending up the coronoid process (white arrows). Individual is AZ 75 T4a (Azapa Valley, Formative Period).
Mandible: mylohyoid line	SPNB	Suggestive	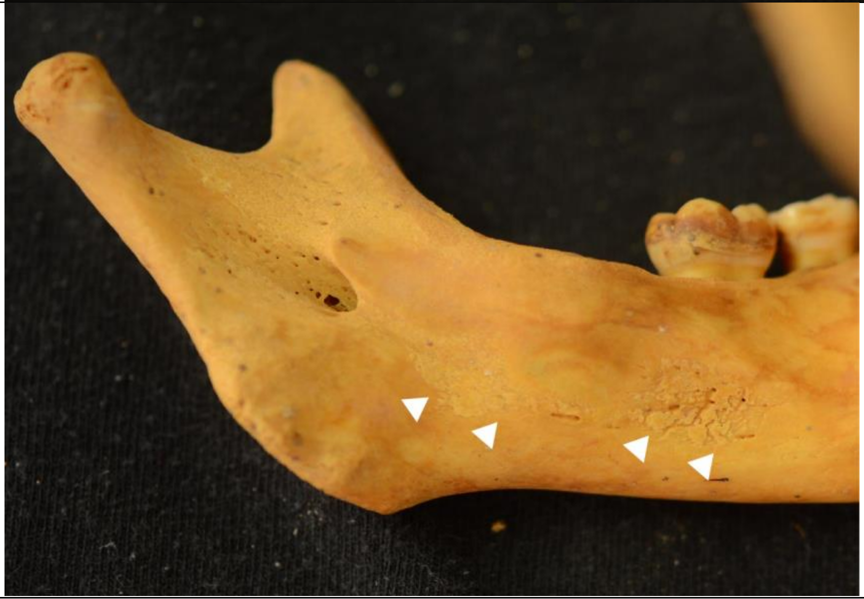	Medial surface of the left half of the mandible of a child (~3-4 years) exhibiting discrete islands of active subperiosteal new bone along the mylohyoid line (white arrows). Individual is AZ 71 CO1 (Azapa Valley, Formative Period).
Occipital: pars basilaris (inferior surface)	Abnormal cortical porosity, SPNB	Suggestive	See Moore and Koon, 2017: Figure 5	NA

Scapulae: supraspinous/infraspinous fosssa	Abnormal cortical porosity, SPNB	Diagnostic	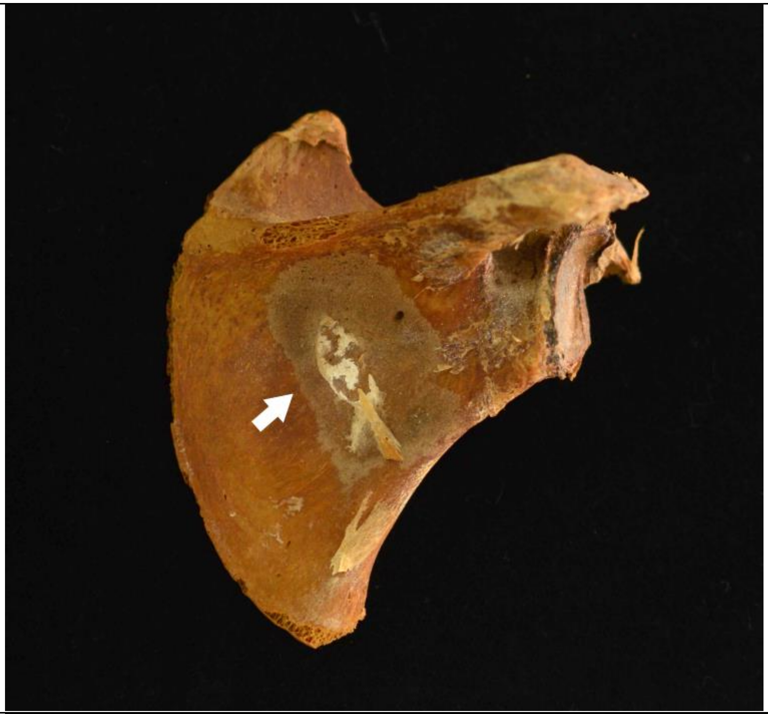	Posterior view of the scapula of a child (~3 years) exhibiting an island of active subperiosteal new bone in the infraspinous fossa. Individual is AZ 75D T8a (Azapa Valley, Formative Period).
Ilium: visceral surface	Abnormal cortical porosity, SPNB, VIs	Suggestive	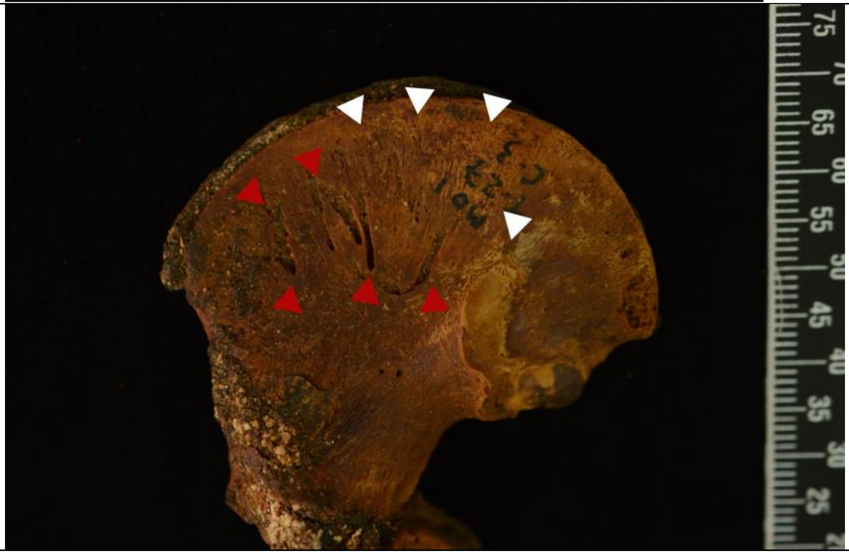	Visceral surface of the right ilium of a child (~6 months) exhibiting patchy, irregular deposition of active subperiosteal new bone (white arrows) and deep vascular channels which penetrate the cortex (red arrows). Dark islands are soft tissue. Individual is Morro 1 T27C3 (Coastal Arica, Archaic Period).
Femur: linea aspera and surrounding region	SPNB	Suggestive	See Buckley et al., 2014: Figure 9	NA
Appendicular skeleton: diaphyses/metaphyses	SPNB (diffuse)	Diagnostic	See Klaus, 2014: Figure 14	NA

Ribs: costochrondral junction	Flaring/swelling	Suggestive	See Schattmann et al, 2016: Figure 4	NA
Ribs: antero-lateral shafts	SPNB	Suggestive	See Snoddy et al., 2017: Figure 4b	NA
Vertebrae: bodies	Biconcavity, osteopenia	Suggestive	See Joffe, 1961: Figure 1	NA
Appendicular skeleton	Bilateral ossified hematomas	Suggestive	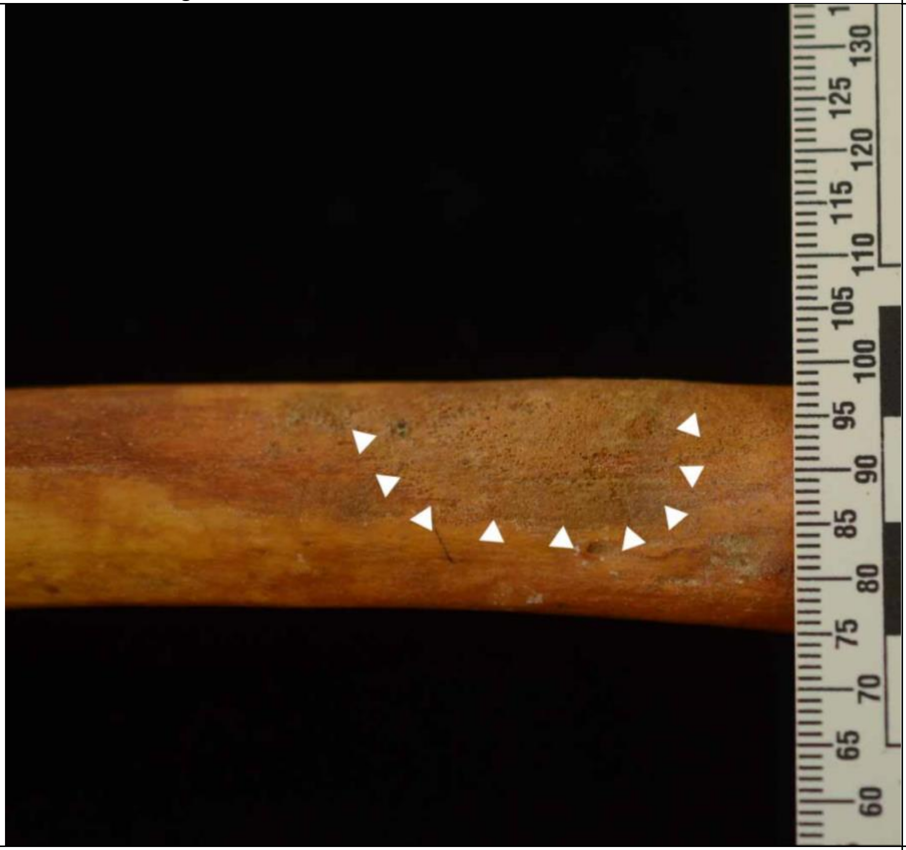	Antero-medial aspect of the left femur of a juvenile (15-20 years) exhibiting an ossified hematoma (white arrows). Individual is AZ 75D T14.
Appendicular skeleton	Metaphyseal cupping/ flaring	Suggestive	See Sprague, 1976: Figures 1 and 2	NA
